# Multiphysics Modelling and Simulation of Thrombolysis via Activated Platelet-Targeted Nanomedicine

**DOI:** 10.1007/s11095-021-03161-2

**Published:** 2022-01-19

**Authors:** Boram Gu, Yu Huang, Emily Louise Manchester, Alun D. Hughes, Simon A. McG. Thom, Rongjun Chen, Xiao Yun Xu

**Affiliations:** 1 Department of Chemical Engineering, Imperial College London, South Kensington Campus, London, UK; 2 School of Chemical Engineering, Chonnam National University, Gwangju, Republic of Korea; 3 Department of Radiology, Shanghai Jiao Tong University Affiliated Sixth People’s Hospital, Shanghai Jiao Tong University School of Medicine, 600 Yi Shan Road, Shanghai, China; 4 Institute of Cardiovascular Science, University College London, London, UK; 5 MRC Unit for Lifelong Health and Ageing at University College London, London, UK; 6 National Heart and Lung Institute, Imperial College London, London, UK

**Keywords:** multiphysics modelling, pharmacodynamics, pharmacokinetics, targeted drug delivery, thrombolysis

## Abstract

**Purpose:**

This study establishes a multiphysics simulation platform for both conventional and targeted thrombolysis using tissue plasminogen activator (tPA). Based on our computational results, the effects of therapeutic parameters on the dynamics of thrombolysis and the risk of side effects are investigated.

**Methods:**

The model extends our previously developed one-dimensional(1D) mathematical models for fibrinolysis by incorporating targeted thrombolysis. It consists of two parts: (i) a coupled mathematical model of systemic pharmacokinetics (PK) and pharmacodynamics (PD) and local PD in a 1D occluded artery, and (ii) a mechanistic model for a targeted thrombolytic system via activated platelet-targeted tPA-loaded nanovesicles (tPA-NV), with model parameters derived from our *in vitro* experiments. A total of 16 therapeutic scenarios are simulated by varying the clot location and composition as well as the dosing regimen with free tPA or tPA-NV.

**Results:**

Our simulation results indicate that tPA-NV offers several advantages over free tPA for thrombolysis. It reduces systemic exposure of tPA, thereby minimising the risk of bleeding complications. Simulations with different tPA-NV doses reveal that tPA-NV at 10% of the recommended dose can be as effective as the standard regimen with the full recommended dose of free tPA, demonstrating the potential of our tPA-NV as a new thrombolytic strategy with a reduced tPA dose. Moreover, faster recanalisation can be achieved with tPA-NV, especially for platelet-rich(or fibrin-poor) clots.

**Conclusions:**

Our simulation platform for thrombolysis with well-tuned model parameters can be used to evaluate and optimise treatment regimens of existing and new thrombolytic therapies via benefit/risk assessment under various therapeutic scenarios.

**Supplementary Information:**

The online version contains supplementary material available at 10.1007/s11095-021-03161-2.

## Introduction

Thrombolytic therapy aims to restore blood flow by dissolving blood clots lodged in blood vessels. If untreated, blood clots can cause catastrophic cardiovascular events such as myocardial infarction, ischaemic stroke and pulmonary embolism (1, 2). Conventional thrombolytic therapy involves intravenous administration of tissue plasminogen activator (tPA), which targets fibrin within blood clots (3, 4). Although this thrombolytic therapy is known to be effective, it can cause severe bleeding complications due to systemic circulation of tPA and its influence on reducing fibrinogen levels. In addition, tPA has a short half-life, approximately 4 to 6 mins (5), which means high doses are needed to maintain therapeutic levels. For example, the dosing regimen recommended by the US Food and Drug Administration (FDA) for the treatment of ischaemic stroke involves administration of 0.9 mg tPA/kg patient weight (10% as a bolus and the remaining 90% as a continuous infusion for an hour) (6). Alternatively, blood clots can be removed through mechanical thrombectomy, which involves the use of a stent retriever (7–13). However, this procedure is more invasive than intravenous thrombolysis, often requires multiple attempts to achieve satisfactory patency and is generally limited to large vessel occlusions (7, 14). Therefore research is ongoing to find alternative approaches, such as exploring novel thrombolytics, new stent-retriever designs, and adjunctive therapies involving intra-arterial catheter-directed thrombolysis (13, 15–18).

One extensively researched strategy towards improved efficacy and reduced side effects involves targeted drug delivery via nanomedicine (19–22). Effective targeted thrombolytic therapy can facilitate prolonged circulation and enhanced delivery of tPA to the clot site while minimising systemic side effects of tPA. We have recently developed a novel targeted thrombolytic system using tPA-loaded nanoparticles (23, 24), one of which comprises lipid-based, fibrinogen-mimicking multi-arm nanovesicles coated with polyethylene glycol (PEG) and cyclic arginine-glycine-aspartic(cRGD) peptide (24). The multifunctional nanovesicle has been proven to have the following advantages: (i) stable and efficient encapsulation of tPA, and (ii) high specificity to α_IIb_β_3_ integrins abundantly expressed on the surface of activated platelets in the clot. Furthermore, many studies have revealed that drug-loaded PEGylated nanomedicine has a prolonged circulation time compared to the direct use of the drug (25–27), although this has not yet been corroborated for our tPA-loaded nanovesicle (tPA-NV) via pharmacokinetic experiments. We also reported a computational model that mimics the tPA-NV system by incorporating the mechanistic understanding of interactions between tPA-NV and activated platelets in the clot under static and flow conditions (24), along with the validated fibrinolysis model (28–30). Our model can simulate tPA leakage in the absence of activated platelets, triggered tPA release dependent on the number of activated platelets in the clot, as well as clot dissolution dynamics by fine-tuning the model parameters using *in vitro* experimental results. We have shown that the model is able to capture the distinctive features of the tPA-NV system. These include delayed lysis initiation in the early phase of drug infusion and accelerated lysis in the later phase, leading to a comparable lysis completion time for free tPA and tPA-NV. This demonstrates the potential use of the computational model in designing a new targeted thrombolytic therapy.

In the present work, we aim to develop an efficient simulation platform for both conventional and targeted thrombolysis. We have previously developed a one-dimensional(1D) thrombolysis model, which combines systemic pharmacokinetics (PK) and pharmacodynamics (PD), transport of tPA and other fibrinolytic proteins, and local fibrinolysis dynamics in the clot (28). This allowed us to investigate the effects of various dosing regimens on recanalisation times and plasma fibrinogen levels, indicative of the treatment outcome and risk of bleeding complications, respectively. Here, we adapt the developed 1D thrombolysis model for the targeted tPA-NV system by incorporating the transport model for tPA-NV and mechanistic model of targeted thrombolysis as additional biochemical reactions (i.e., tPA leakage and release kinetics) in the coupled PKPD model. In addition, the temporal evolution of clot properties with the extent of lysis is modified to account for the presence of activated platelets in the clot. The new 1D model is used to compare the efficacy and risk of tPA-NV with conventional tPA for thrombolysis treatment in a variety of clinically relevant scenarios. It is hoped that our model can help optimise the dosing protocol to facilitate potential clinical translation of the new tPA-NV system.

## Methods

### Overview of the Targeted Thrombolytic System and Modelling Strategy

**Fig. 1:**
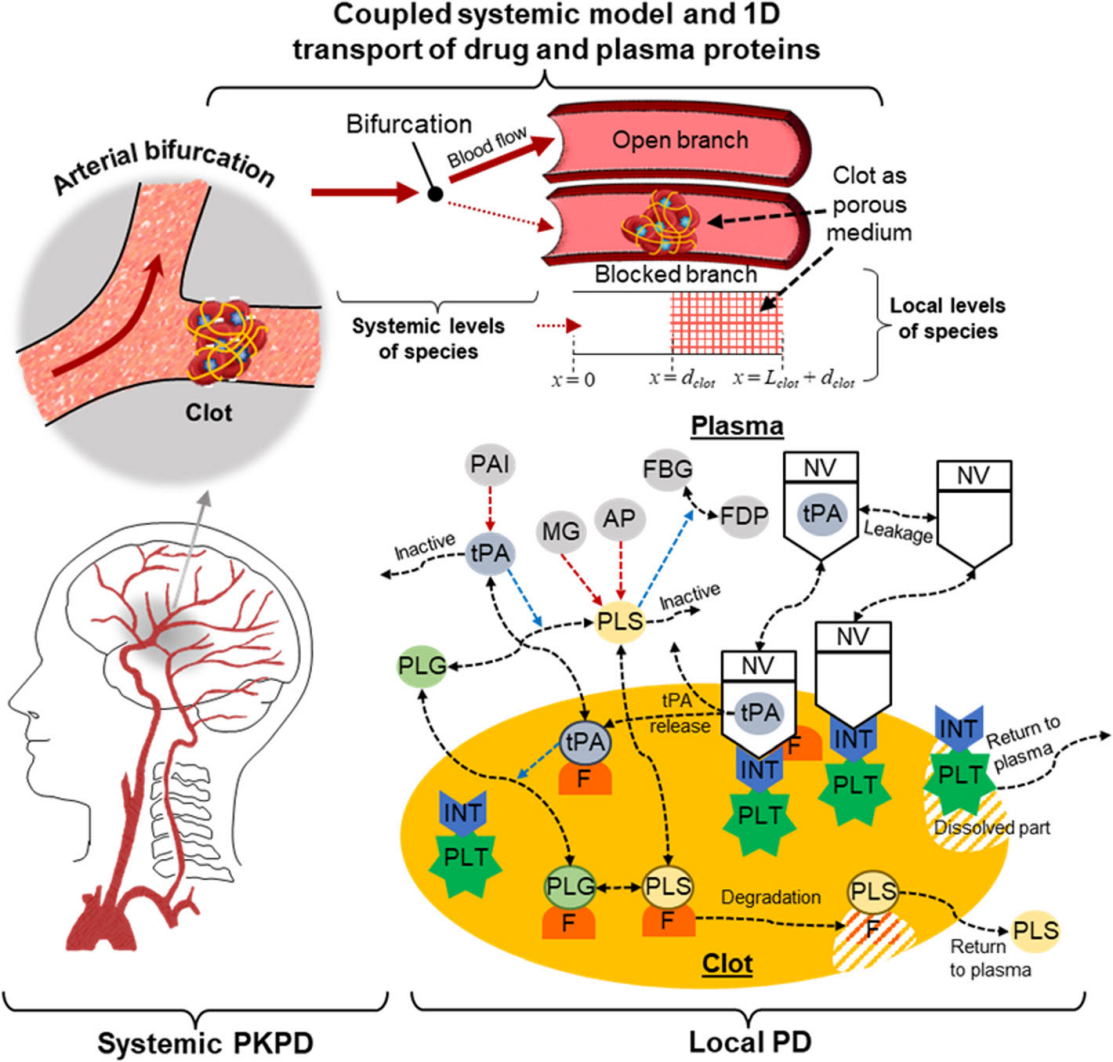
An overview of the targeted thrombolytic system via the novel multifunctional NV in our previous work (24) and the key components of our multiphysics model: systemic PKPD, local PD in the clot site and 1D transport of NV and plasma proteins. tPA: tissue plasminogen activator; PLG: plasminogen; PLS: plasmin; AP: α_2_-antiplasmin; MG: α_2_-macroglobulin; FBG: fibrinogen; FDP: fibrin degradation product; PAI: plasminogen activator inhibitor-1; PLT: activated platelets; INT: α_IIb_β_3_ integrins; F: fibrin fibre binding sites; NV (with tPA in it): tPA-NV, NV (empty): empty NV following tPA leakage or release. Blue dashed arrows: enzymatic action on conversion reactions, red dashed arrows: inhibition or inactivation, black dashed arrows: binding/unbinding or reversible/irreversible conversion reaction.

An overview of the targeted thrombolytic system (24) is illustrated in Fig. 1. Briefly, tPA-loaded nanovesicles (tPA-NV) bind with α_IIb_β_3_ integrins (INT) expressed on the surface of activated platelets (PLT) in the clot. Upon binding, tPA is released and rapidly binds to fibrin binding sites in the fibrin fibre network. In addition, there is a small amount of tPA leakage from unbound NV into the plasma phase due to the concentration gradient between the tPA level inside the NV and its surroundings. Once tPA reaches its therapeutic level in the plasma, fibrinolysis occurs. Full details of fibrinolytic kinetics can be found in our previous work (24, 28–30). From here onwards, NV is used interchangeably with tPA-NV for brevity and NV following tPA release and leakage is referred to as NV_emp_.

The computational model comprises three parts, as shown in Fig. 1. Firstly, the systemic PKPD model predicts plasma levels of NV, tPA and other fibrinolytic proteins described by a compartmental model. Secondly, the local PD model includes a number of biochemical reaction kinetics, i.e., tPA leakage, binding/unbinding between NV and INT and triggered tPA release in addition to the fibrinolytic reaction kinetics. Finally, the convective and diffusive transport of species in a clotted vessel is coupled with the aforementioned systemic PKPD and local PD models.

### Model Equations

The modelling strategy adopted in this work is to combine two models developed in our previous work: systemic PKPD-local PD modelling in (28) and targeted thrombolysis kinetics in (24). Here we only present the fundamental model equations describing each part shown in Fig. 1. A full list of equations and model details can be found either in Section A of the Supporting Information or our previous publications (24, 28).

#### Systemic PKPD Model

A single compartmental model is used to resolve temporal systemic concentrations of tPA and plasma proteins by accounting for tPA administration, hepatic clearance, systemic secretion and plasma reactions.

1 $$ \frac{d{C}_{tPA, sys}(t)}{dt}=\frac{I_{tPA}(t)}{V_cM{w}_{tPA}}-{k}_{el, tPA}{C}_{tPA, sys}(t)+{S}_{tPA}(t)+{r}_{tPA}^{plasma}(t) $$

2 $$ \frac{d{C}_{i, sys}(t)}{dt}=-{k}_{el,i}{C}_{i, sys}(t)+{r}_i^{plasma}(t)+{S}_i(t),\kern0.5em \mathrm{at}\ i=\mathrm{PLG},\mathrm{PLS},\mathrm{AP},\mathrm{AP}\hbox{-} \mathrm{PLS},\mathrm{FBG},\mathrm{MG}\ \mathrm{and}\ \mathrm{PAI} $$

where *t* is time, *C* the systemic concentration in μM, *I* the infusion rate in mg/s, *V*_*c*_ the volume of plasma, *Mw* the molecular weight, *k*_*el*_ the elimination rate constant, *S* the generation rate and *r* the reaction source term and the subscript *i,sys* denotes systemic species *i*. For NV administration, temporal concentrations of NV (*C*_*NV*_) and NV after tPA leakage (*C*_*NVemp*_) are resolved by:

3 $$ \frac{d{C}_{NV, sys}(t)}{dt}=\frac{I_{NV}(t)}{V_cM{w}_{NV}}-{k}_{el, NV}{C}_{NV, sys}(t)+{r}_{NV}^{plasma}(t) $$

4 $$ \frac{d{C}_{NVemp, sys}(t)}{dt}=-{k}_{el, NV}{C}_{NVemp, sys}(t)+{r}_{NVemp}^{plasma}(t) $$

It is assumed that tPA-loaded NV (NV) and empty NV (NV_emp_) have the same clearance rate. Depending on the type of drugs, either *I*_*tPA*_(*t*) or *I*_*NV*_(*t*) is set to 0. The reaction source terms for each component in Eqs. (1) to (4) can be calculated by combining relevant individual reaction terms presented in Section A.2 of the Supporting Information.

#### Coupled Transport of Species and Local PD Model

Flow in the thrombosed artery is assumed to be governed by the continuity equation for incompressible flow in Eq. (5) and Darcy’s law for flow through a porous medium in Eq. (6),

5 $$ \frac{\partial Q(t)}{\partial x}=0 $$

6 $$ Q(t)=\frac{\varDelta {P}_x{L}_{clot, rem}(t)A}{\mu {\int}_0^L\frac{R_{clot, tot}\left(t,x\right)}{\varepsilon \left(t,x\right)}\; dx} $$

where *x* is the axial coordinate, *Q* the volumetric flowrate along the occluded artery, Δ*P*_*x*_ the pressure drop per unit length across the clot, *L*_*clot,rem*_ the remaining fibrin clot length, *A* the cross-sectional area of the artery, *μ* the blood viscosity, *R*_*clot,tot*_ the total clot resistance, *ε* the porosity and *L* the length of the clot. Equations to calculate variables dependent on the progression of clot lysis can be found in Section A of the Supporting Information, which includes: the total resistance of the clot (*R*_*clot,tot*_), the resistance of fibrin fibre network (*R*_*clot,FBR*_) and activated platelets (*R*_*clot,PLT*_) present in the clot, the volume fraction of fibrin fibre network (*ϕ*_*FBR*_) and activated platelets (*ϕ*_*PLT*_) in the clot and the local porosity (*ε*) in the occluded artery.

**Fig. 2:**
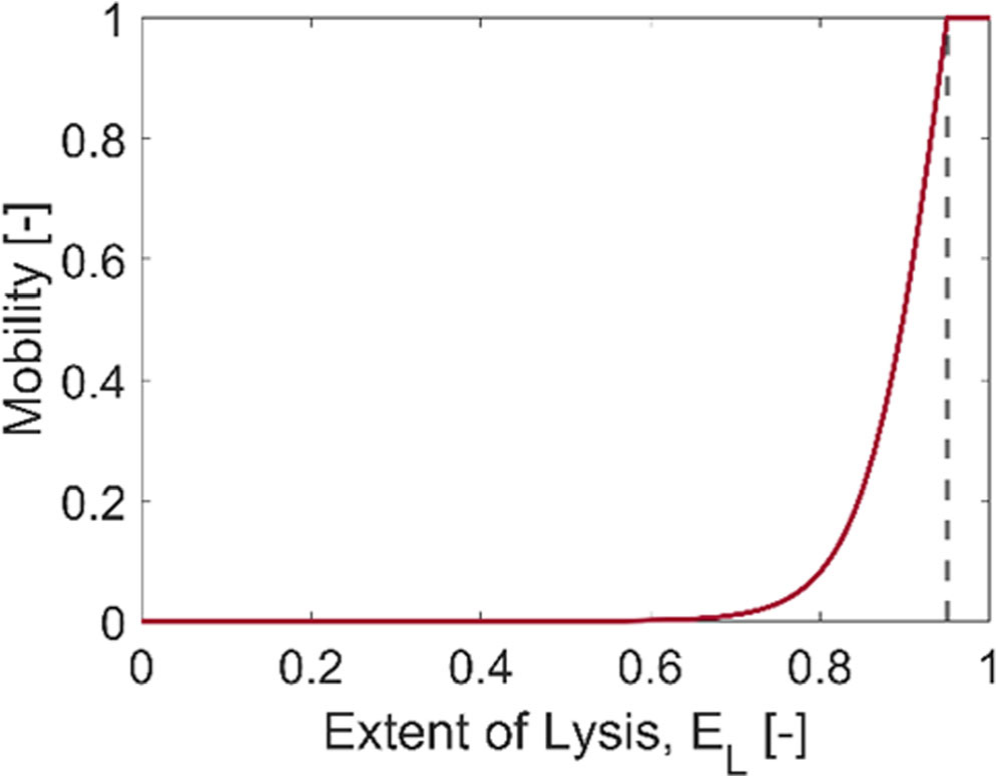
Mobility as a function of the extent of lysis when *m* = 10 and *E*_*L,crit*_ = 0.95.

The transport of unbound species is governed by:

7 $$ {\displaystyle \begin{array}{c}\frac{\partial {C}_j\left(t,x\right)}{\partial t}=-\frac{\partial u(t){C}_j\left(t,x\right)}{\partial x}+{D}_j\frac{\partial^2{C}_j\left(t,x\right)}{\partial {x}^2}+{r}_j^{tot}\left(t,x\right),\\ {}\kern4.199998em \mathrm{for}\;j=\mathrm{tPA},\mathrm{PLG},\mathrm{PLS},\mathrm{FBG},\mathrm{AP},\mathrm{AP}\hbox{-} \mathrm{PLS},\mathrm{MG},\mathrm{PAI},\mathrm{NV},{\mathrm{NV}}_{\mathrm{emp}}\end{array}} $$

where *u* is the Darcy velocity (see Section A.3 in the Supporting Information for details on how to calculate this parameter) and *D* is the diffusion coefficient. The superscript *tot* denotes the reaction source term, i.e., the sum of both the plasma phase and clot-bound phase. The activated platelets within the clot can move out of the clot depending on the extent of fibrin degradation, i.e., the extent of lysis, *E*_*L*_. Total activated platelets, NV-bound activated platelets and empty NV-bound activated platelets are obtained by the modified convection-diffusion-reaction equation in Eq. (8).

8 $$ {\displaystyle \begin{array}{c}\frac{\partial {C}_k\left(t,x\right)}{\partial t}=-M(t)\frac{\partial u(t){C}_k\left(t,x\right)}{\partial x}+M(t)\cdot {D}_{PLT}\frac{\partial^2u(t){C}_k\left(t,x\right)}{\partial {x}^2}+{r}_k^{tot}\left(t,x\right),\\ {}\kern10.67999em \mathrm{for}\ k=\mathrm{PLT}\hbox{-} \mathrm{tot},\mathrm{PLT}\hbox{-} \mathrm{NV},\mathrm{PLT}\hbox{-} {\mathrm{NV}}_{\mathrm{emp}}\end{array}} $$

9 $$ M(t)=\left\{\begin{array}{c}1,\kern0.75em \mathrm{for}\kern0.5em {E}_L>{E}_{L, crit}\\ {}1-\tanh \left(m\left(1-\frac{E_L}{E_{L, crit}}\right)\right),\mathrm{for}\ {E}_L\le {E}_{L, crit}\end{array}\right. $$

where *M* is the mobility coefficient determined by *E*_*L*_ as in Eq. (9) and *m* is an arbitrary constant that determines the slope of the mobility curve at the critical extent of lysis *E*_*L,crit*_, shown in Fig. 2. All simulations presented here were performed with *m* = 10.

The concentration of activated platelets and free INT sites in the clot is calculated as:

10 $$ {C}_{PLT\hbox{-} free}\left(t,x\right)={C}_{PLT, tot}\left(t,x\right)-{C}_{PLT\hbox{-} NV}\left(t,x\right)-{C}_{PLT\hbox{-} N{V}_{emp}}\left(t,x\right) $$

11 $$ {C}_{INT,k}\left(t,x\right)={N}_{INT}{C}_k\left(t,x\right),\mathrm{for}\ k=\mathrm{PLT}\hbox{-} \mathrm{free},\mathrm{PLT}\hbox{-} \mathrm{NV},\mathrm{PLT}\hbox{-} {\mathrm{NV}}_{\mathrm{emp}} $$

where *N*_*INT*_ is the average number of integrins expressed on an activated platelet. The content of activated platelets in the clot that varies over time is defined using the extent of activated platelets, *E*_*PLT*_:

12 $$ {E}_{PLT}\left(t,x\right)=\frac{C_{PLT, tot}\left(t,x\right)}{C_{PLT,0}} $$

where *C*_*PLT,0*_ is the initial concentration of activated platelets in the clot. The concentrations of bound tPA, PLG and PLS with the fibrin fibre (tPA-F, PLG-F and PLS-F, respectively) in the clot and lysed fibrin binding sites with PLS still present (PLS-F_lysed_) are obtained by solving the following equations:

13 $$ \frac{\partial {n}_h\left(t,x\right)}{\partial t}={r}_h^{clot}\left(t,x\right)\ \mathrm{for}\ \mathrm{h}=\mathrm{tPA}\hbox{-} \mathrm{F},\mathrm{PLG}\hbox{-} \mathrm{F},\mathrm{PLS}\hbox{-} \mathrm{F},\mathrm{PLS}\hbox{-} {\mathrm{F}}_{\mathrm{lysed}} $$

14 $$ \frac{\partial {n}_{FBR}\left(t,x\right)}{\partial t}=-{r}_{deg}\left(t,x\right) $$

where *n*_*h*_ is the concentration of bound phase species and *n*_*FBR*_ is the total fibrin binding sites. Using the calculated *n*_*FBR*_ by Eq. (14), the extent of lysis *E*_*L*_ is calculated by Eq. (15), which is used to determine the mobility of activated platelets in the clot, as in Eq. (9),

15 $$ {E}_L=1-\frac{n_{tot}}{n_{tot,0}} $$

where *n*_*tot,0*_ is the initial concentration of fibrin binding sites.

### Solution Procedure

#### Initial and Boundary Conditions

In order to solve the ordinary and partial differential equations in Eqs. (1)–(4), (7)–(8) and (13)–(14), initial and inlet concentrations of species should be provided. Temporal systemic concentrations are obtained by solving Eqs. (1) to (4) using the following initial conditions.

For tPA, NV, NV_emp_, PLG, PLS, AP, AP-PLS, FBG, MG and PAI:

16 $$ {C}_i(t)={C}_{i,0}\ \mathrm{at}\ t=0,\mathrm{for}\ i=\mathrm{species} $$

17 $$ {C}_i\left(t,x\right)={C}_{i,0}\ \mathrm{at}\ t=0\ \mathrm{and}\ x\ge 0,\mathrm{for}\ i=\mathrm{species} $$

18 $$ {C}_i\left(t,x\right)={C}_{i, sys}(t)\ \mathrm{at}\ t>0\ \mathrm{and}\ x=0,\mathrm{for}\ i=\mathrm{species} $$

19 $$ \frac{{\partial C}_i\left(t,x\right)}{\partial x}=0\ \mathrm{at}\ t>0\ \mathrm{and}\ x={d}_{clot}+{L}_{clot},\mathrm{for}\ i=\mathrm{species} $$

where *d*_*clot*_ is the distance between the bifurcation and clot front and *L*_*clot*_ is the clot length.

For NV administration, an additional condition at the clot front, described by Eq. (20), is applied to deal with the sudden elevation of tPA level due to the triggered release upon the contact of NV with PLT.

20 $$ \frac{\partial {C}_{tPA}\left(t,x\right)}{\partial x}=0\ \mathrm{at}\ t>0\ \mathrm{and}\ x=\mathrm{clot}\kern0.17em \mathrm{front}\ \left(\varepsilon <1\right) $$

Imposing Eq. (20) at the clot front ensures that the spatial profile of tPA concentration is continuous and differentiable throughout the domain for numerical robustness.

For PLT, PLT-NV and PLT-NV_emp_:

21 $$ {C}_{PLT\hbox{-} tot}\left(t,x\right)\left\{\begin{array}{c}{C}_{PLT,0},\kern0.5em \mathrm{for}\ {d}_{clot}\le x\le {d}_{clot}+{L}_{clot}\\ {}0,\kern0.5em \mathrm{otherwise}\end{array}\right.\ \mathrm{at}\ t=0 $$

22 $$ {C}_{PLT\hbox{-} NV}\left(t,x\right)=0\kern0.5em \mathrm{at}\ t=0\ \mathrm{and}\ x\ge 0 $$

23 $$ {C_{PLT\hbox{-} NV}}_{emp}\left(t,x\right)=0\kern0.5em \mathrm{at}\ t=0\ \mathrm{and}\ x\ge 0 $$

The initial concentration of activated platelets in the clot *C*_*PLT,0*_ is calculated from the initial volume fraction of activated platelets (*ϕ*_*f,0*_) in the clot. It is assumed that activated platelets are homogeneously distributed within the clot.

For tPA-F, PLG-F, PLS-F and FBR:

24 $$ {n}_m\left(t,x\right)=0\kern0.5em \mathrm{at}\ t=0\ \mathrm{and}\ x\ge 0,\mathrm{for}\ m=\mathrm{tPA}-\mathrm{F},\mathrm{PLG}-\mathrm{F},\mathrm{PLS}-\mathrm{F} $$

25 $$ {n}_{FBR}\left(t,x\right)=\left\{\begin{array}{c}{n}_{FBR,0},\kern0.5em \mathrm{for}\ {d}_{clot}\le x\le {d}_{clot}+{L}_{clot}\\ {}\begin{array}{cc}0,& \mathrm{otherwise}\end{array}\end{array}\ \mathrm{at}\ t=0\right. $$

where *n*_*FBR,0*_ is the initial number of fibrin binding sites, which is estimated using the radius of fibrin fibres. The initial concentration of fibrin binding sites is assumed to be uniform over the clot region.

#### Model Integration and Numerical Details

Model solutions are obtained by an in-house code programmed in MATLAB R2019b (The MathWorks, Inc., Natick, MA, United States). A total of 10 ordinary differential equations shown in Eqs. (1)–(4) are solved for the temporal systemic concentrations of tPA, NV, NV_emp_, PLG, PLS, AP, AP-PLS, FBG, MG and PAI and a total of 18 partial differential equations shown in Eqs. (7)–(8) and (13)–(14) are solved for temporal and spatial concentrations of tPA, NV, NV_emp_, PLG, PLS, AP, AP-PLS, FBG, MG, PAI, PLT-free, PLT-NV, PLT-NV_emp_, tPA-F, PLG-F, PLS-F, PLS-F_lysed_ and FBR. Eqs (1)–(4) are solved first and the obtained solutions are used as inlet conditions for Eqs. (7)–(8) and (13)–(14).The same numerical procedure for model integration is employed as in our previous work and can be found in (28).

### Simulation Scenarios and Model Parameters

Sixteen simulations are performed in this study (Table 1): the first 12 scenarios are designed to compare the effectiveness of free tPA and NV under different clot conditions (i.e,. the clot position and composition). The selected scenarios are based on the values reported in clinical studies: composition and porosity in (31), clot position (32) and size (33, 34). Scenarios 1 and 2 represent more distal clots, while Scenarios 5 and 6 correspond to platelet-rich clots compared to the other simulated scenarios. Scenarios 7 to 12 have a clot of higher overall porosity (*ε*_*0*_ = 0.98) with different platelet and fibrin contents than Scenarios 1 to 6 (*ε*_*0*_ = 0.95). For these scenarios, only the coupled species transport with the local PD model is solved with constant inlet concentrations of plasma proteins, i.e. without the systemic PKPD model. The inlet concentration of tPA is chosen to be 0.035 μM, the therapeutic level typically achieved during a continuous infusion in the recommended dosage regimen for the treatment of ischaemic stroke (28, 30). For the simulation scenarios with NV, the inlet NV concentration equivalent to 0.035 μM of tPA (0.035 μM/*v*_*rel*_, *v*_*rel*_ the ratio of encapsulated tPA to NV) is chosen for a fair comparison.

**Table 1:** List of simulation scenarios. Here NV stands for tPA-loaded nanovesicle

No.	Description	*d*_*clot*_ [mm]	*L*_*clot*_ [mm]	*ϕ*_*FBR,0*_ [−]	*ϕ*_*PLT,0*_ [−]	Drug	Inlet
S1	Distal dense PLT-poor clot	2	4	0.02	0.03	tPA	Constant
S2	Distal dense PLT-poor clot	2	4	0.02	0.03	NV	Constant
S3	Proximal dense PLT-poor clot	0	4	0.02	0.03	tPA	Constant
S4	Proximal dense PLT-poor clot	0	4	0.02	0.03	NV	Constant
S5	Proximal dense PLT-rich clot	0	4	0.01	0.04	tPA	Constant
S6	Proximal dense PLT-rich clot	0	4	0.01	0.04	NV	Constant
S7	Proximal coarse clot	0	4	0.01	0.01	tPA	Constant
S8	Proximal coarse clot	0	4	0.01	0.01	NV	Constant
S9	Proximal coarse PLT-poor clot	0	4	0.015	0.005	tPA	Constant
S10	Proximal coarse PLT-poor clot	0	4	0.015	0.005	NV	Constant
S11	Proximal coarse PLT-rich clot	0	4	0.005	0.015	tPA	Constant
S12	Proximal coarse PLT-rich clot	0	4	0.005	0.015	NV	Constant
S13	Standard regimen Recommended dose: 0.9 mg/kg	0	4	0.02	0.03	tPA	Systemic PKPD
S14	Standard regimen Recommended dose	0	4	0.02	0.03	NV	Systemic PKPD
S15	Bolus only 10% recommended dose	0	4	0.02	0.03	NV	Systemic PKPD
S16	Standard regimen 50% recommended dose	0	4	0.02	0.03	NV	Systemic PKPD

Scenarios 13 to 16 are designed to study the effects of different dosing regimens compared to the standard treatment protocol with free tPA. For these scenarios, the systemic PKPD model is solved, and its solutions are used as inlet conditions for the species transport model with the local PD model. The recommended regimen with free tPA for the treatment of ischaemic stroke is selected as a reference case: a total tPA dose of 0.9 mg/kg is intravenously administered with 10% of the total dose as a bolus and the remaining 90% as a continuous infusion over 1 h (Scenario 13). For Scenario 14, the equivalent NV amount is given with the same dosing regimen as Scenario 13. Different dosing regimens are simulated for NV in Scenarios 15 and 16; a total dose of 0.09 mg/kg (10% of the recommended dose) as a bolus in Scenario 15 and a total dose of 0.45 mg/kg (50% of the recommended dose) with the standard dosing protocol (a combination of bolus and continuous infusion) in Scenario 16.

Values for the model parameters are the same as in our previous studies (24) with a modification of NV and PLT diffusivity. Diffusivity values for the NV and platelets should be carefully chosen since diffusion-dominated transport is anticipated in an occlusive clot, unlike in our previous work where non-occlusive clots were simulated (24). Lipid nanovesicles are reported to have diffusivity in the range of 0.126 × 10^−12^ to 1.689 × 10^−12^ m^2^/s depending on their size (mean diameter of 184 nm to 216 nm, a similar range to our NV) (35). Therefore, a diffusivity value of 1.6 × 10^−12^ m^2^/s is chosen for the simulations. PLT diffusivity was estimated by the Stokes-Einstein equation (36) and is chosen to be 3.1 × 10^−14^ m^2^/s. The half-life time of the developed NV is a new parameter needed for the systemic PKPD model and is chosen to be 133 min, as found in the literature for tPA-loaded PEGylated liposomes (25).

Simulation conditions for all 16 scenarios are summarised in Table 1, and other conditions used in common for all the scenarios are listed in Section B of the Supporting Information, along with kinetics parameters, initial concentrations and other model parameters.

## Results and Discussion

Here, we present recanalisation times for all simulated scenarios and then several selected model variables that can help us to gain valuable insights into the complex thrombolysis dynamics. Additional results of the spatial and temporal distributions solved by the model can also be found in Section C of the Supporting Information.

### Summary of all Simulated Scenarios

**Fig. 3:**
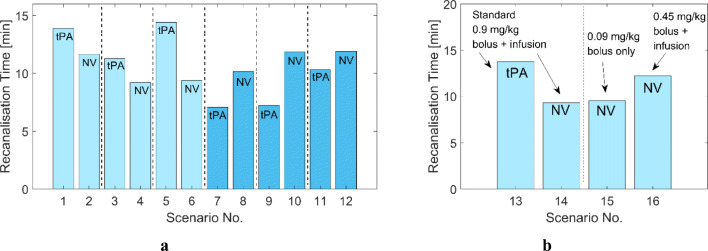
Recanalisation times for the simulated scenarios. (**a**) S1 to S12 with constant inlet concentrations of plasma proteins and drug, Light blue bars: Dense clot, Dark blue bars: coarse clot, (**b**) S13 to S16 for dense clots with time-varying inlet concentrations of plasma proteins and drug for different dosage regimens. The text tPA and NV on each bar represents the type of drug used in each scenario: free tPA vs. tPA-loaded nanovesicle.

Complete dissolution of the clots is achieved in all simulated scenarios with completion time at 7–15 min, as displayed in Fig. 3, which is at the lower end of the reported range of reperfusion time at 23 ± 16 min (37). In terms of computation time, each simulation takes less than a few minutes to complete using a standard computer. Simulation results reveal that compared to conventional tPA therapy, our targeted tPA-loaded NV is more effective in dissolving dense clots (S1–6 and S13–14), achieving faster recanalisation, but less effective in treating coarse clots (S7–12). This suggests that our tPA-loaded NV might be more suitable for clots that contain a sufficient level of activated platelets in order to effectively trigger the release of tPA. Also, it is shown that targeted thrombolysis (S14-S16), regardless of the dose of tPA-loaded NV, takes less time for complete dissolution than conventional tPA therapy (S13) in the simulated clots. Further analyses are included in the subsequent sections separately for S1–12 with the local PD model and S13–16 with the coupled systemic PKPD and local PD model.

### Simulation Results with Constant Inlet Concentrations (S1 to S12)

#### Temporal and Spatial Distributions

**Fig. 4:**
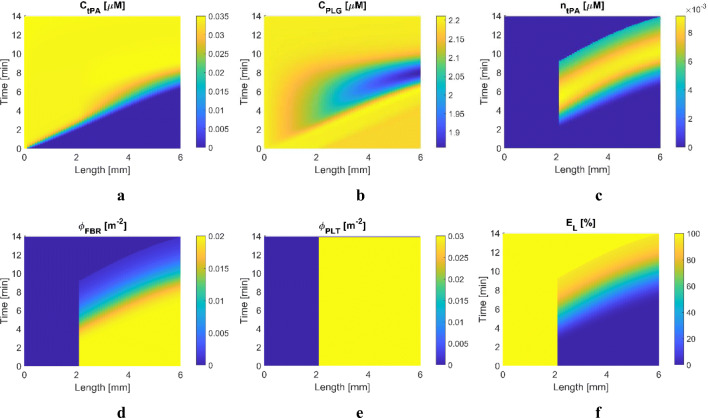
Simulation results for Scenario 1: a 4-mm clot with *ϕ*_*FBR*_ = 0.02 and *ϕ*_*PLT*_ = 0.03 is located 2 mm away from the entrance of the blocked artery and is treated with free tPA. Spatial (horizontal axis) and temporal (vertical axis) variations of key variables, (**a**) plasma phase tPA concentration, (**b**) plasma phase PLG concentration, (**c**) FBR-bound phase tPA concentration, (**d**) fibrin fibre volume fraction, (**e**) activated platelets volume fraction, (**f**) the extent of lysis.

**Fig. 5:**
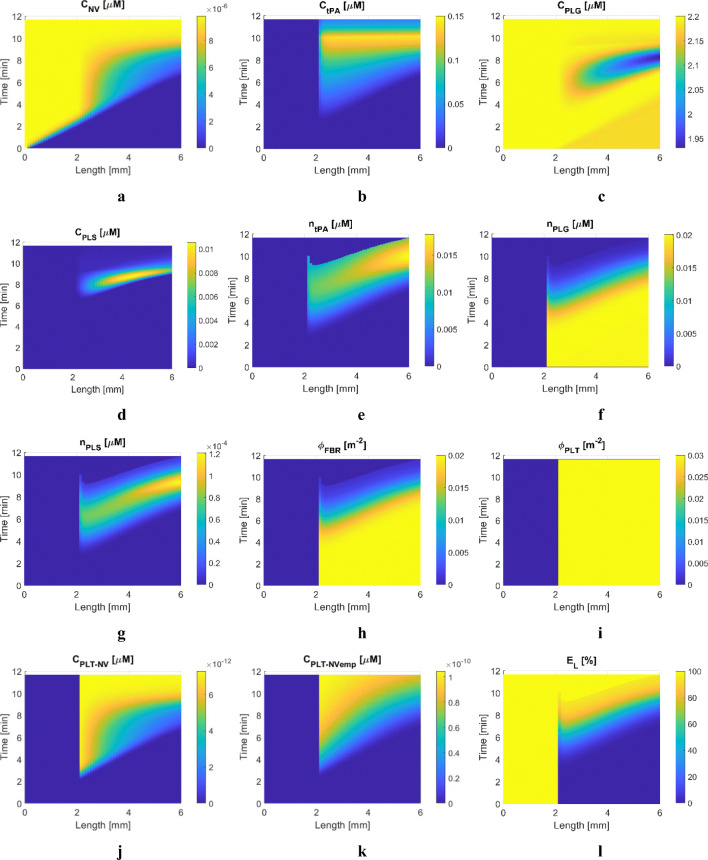
Simulation results for Scenario 2: a 4-mm clot with *ϕ*_*FBR*_ = 0.02 and *ϕ*_*PLT*_ = 0.03 is located 2 mm away from the entrance of the blocked artery and is treated with tPA-loaded NV. Spatial (horizontal axis) and temporal (vertical axis) variations of key variables, (**a**) plasma phase tPA-loaded NV concentration, (**b**) plasma phase tPA concentration, (**c**) plasma phase PLG concentration, (**d**) plasma phase PLS concentration, (**e**) FBR-bound phase tPA concentration, (**f**) FBR-bound phase PLG concentration, (**g**) FBR-bound phase PLS concentration, (**h**) FBR volume fraction, (**i**) PLT volume fraction, (**j**) PLT-bound NV concentration, (**k**) PLT-bound empty NV concentration, (**l**) extent of fibrinolysis.

Simulation results of S1 and S2 are presented in Figs. 4 and 5, respectively. For S1 with free tPA, temporal and spatial results shown in Fig. 4 are similar to those in our previous study (28). Nonetheless, results are included here to confirm the consistency in simulation results after incorporating PLT in the adapted model. The transport rate of free tPA in the proximal clot-free region (*x* = 0 to 2 mm) appears to be relatively linear, compared to that within the clot region where tPA reaches the clot front face at around 2 min, shown in Fig. 4a. As fibrinolysis progresses, PLG becomes depleted, as shown in Fig. 4b, resulting in slower lysis at the later stage of treatment. It has been reported that a low PLG level can lead to reduced lysis efficacy (38). Fast conversion of PLG in abundant tPA would initially improve the lysis rate at the expense of reduced lytic efficacy and increased risk of bleeding complications caused by lowered systemic FBG level, which is demonstrated later with systemic PKPD results.

While the fibrin volume fraction gradually decreases over time (Fig. 4d), the volume fraction of activated platelets hardly varies, as can be seen in Fig. 4e. This is because the mobility of PLT is determined by the hyperbolic tangent function in the current model so that the mobility coefficient increases with increasing *E*_*L*_ and finally reaches unity when *E*_*L*_ > *E*_*L,crit*_; that is, PLT can only leave the clot when the extent of fibrinolysis is sufficiently high (around 0.7 as shown in Fig. 2) and becomes fully mobile at *E*_*L*_ > 0.95. Furthermore, even if the mobility coefficient is non-zero inside the clot, PLT has a very small diffusivity (3.1 × 10^−14^ m^2^/s) and the permeation velocity is extremely low (< 4 × 10^−5^ m/s initially). In the occlusive clots considered in this work, the movement of PLT is highly limited until the clot is completely dissolved, with a breakthrough path being established. Therefore, PLT remains largely inside the clot and would eventually be released when the entire clot disappears. This is a limitation of the current model, which will be addressed later.

Figure 5 displays spatial and temporal variations of selected species concentrations and additional variables relevant to the progress of clot lysis for S2, where the same clot as in S1 is treated with tPA-loaded NV. The level of NV steadily increases over time along the axial direction due to the permeation flow through the blocked artery (Fig. 5a). There is no free tPA in the proximal clot-free region (*x* = 0 to 2 mm) and tPA is only released within the clot region after NV binds with INT in the clot (Fig. 5b). Due to the triggered release of tPA upon contact with PLT, the level of free tPA gradually rises and then peaks at 0.15 μM towards the final phase of clot lysis at around 10 min. The tPA level in S2 is much higher than S1 on average, i.e., up to 0.15 μM in S2 and up to 0.035 μM in S1. This is because there is still PLT present in the clot (Fig. 5h and i for *ϕ*_*FBR*_ and *ϕ*_*PLT*_, respectively) with a high level of NV and PLT-NV, which leads to a continuous tPA release and build-up of released tPA in the clot region due to slow transport into the remaining clot.

In addition, the high concentration of tPA leads to fast conversion of PLG to PLS in the plasma and bound phase, as shown in Figs. 5c-g, which is also observed in S1. There are excessive amounts of bound tPA in the later stage of lysis in Fig. 5e, while low levels of bound PLG and conversely high levels of bound PLS are maintained only for a short time, shown in Figs. 5f-g. This suggests that the high level of bound tPA does not directly contribute to further accelerated lysis of the fibrin fibre network because bound PLS is the main species to cleave the fibrin fibre network. Therefore, an optimal level of tPA should be found to ensure the conversion of PLG to PLS at an adequate rate.

Interesting lysis patterns are observed in S2, especially near the clot front, which are not found in S1. As can be seen in Fig. 5l, the extent of fibrin lysis at the clot front face (*x* = 2 mm) is slightly lower than that in the immediate downstream region. As this phenomenon persists for a few minutes, complete lysis is achieved from the inner part of the clot at 9.5 min (Fig. 5l). This is because blood flow is modelled in one direction along the clot length. Consequently, the convective transport of tPA is always from the clot front towards the end once NV releases tPA into the plasma phase. This results in a slightly lower tPA concentration at the clot front (Fig. 5b). As explained in S1, the spatial distribution of the total PLT hardly changes in time until the clot end is completely dissolved, shown in Fig. 5i, although the NV-bound and NV_emp_-bound PLT levels change spatially and temporally (Fig. 5j and k).

**Fig. 6:**
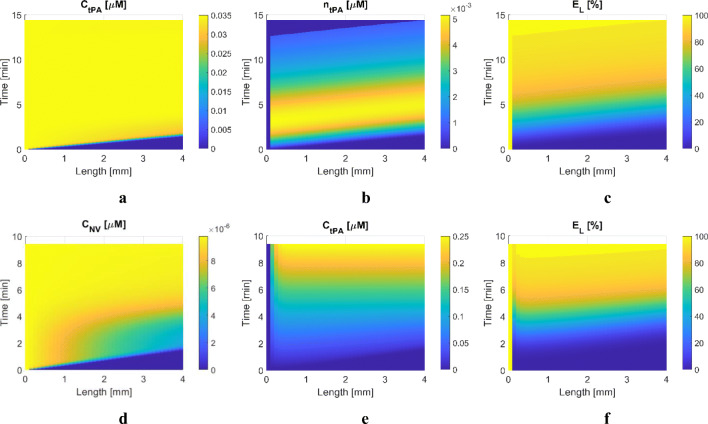
Simulation results for Scenarios 5 and 6: a 4-mm clot with *ϕ*_*FBR*_ = 0.01 and *ϕ*_*PLT*_ = 0.04 is located at the entrance of the blocked artery and is treated with tPA (S5) and tPA-loaded NV (S6). Spatial (horizontal axis) and temporal (vertical axis) variations of key variables, (**a**) plasma phase tPA concentration in S5, (**b**) FBR-bound phase tPA concentration in S5, (**c**) the extent of fibrinolysis in S5, (**d**) plasma phase NV concentration in S6, (**e**) free phase tPA concentration in S6, (**f**) the extent of fibrinolysis in S6.

In Fig. 6, concentrations of free tPA and FBR-bound tPA and the extent of lysis are displayed for S5 and S6 where a relatively PLT-rich clot is simulated. Although the total volume fraction of the clot in S5 is set to be identical to S1 (*ϕ*_*FBR*_ + *ϕ*_*PLT*_ = 0.05), the transport of free tPA appears to be faster in the PLT-rich clot than in the PLT-poor clot. This is attributed to the different total clot resistance – the sum of resistances imposed by PLT and FBR; for the same volume fraction, the PLT resistance is calculated to be 2–3 orders of magnitude smaller than the FBR resistance, as described in Section C2 in the Supporting Information. Therefore, clot permeability is 2–3 orders of magnitude larger in S5 than in S1, which leads to an increased flowrate and improved transport rate of tPA across the clot.

For S6 with a PLT-rich clot, free tPA distribution in Fig. 6e is different from S2 (Fig. 5b), although lysis patterns are similar to S2 (Fig. 6f and Fig. 5l). As mentioned earlier, the PLT-rich clot has higher permeability than the PLT-poor clot, and consequently, a faster transport rate of NV and released tPA is achieved. Furthermore, due to the higher PLT concentration, more tPA can be released from NV in the PLT-rich clot, which leads to a higher concentration of free tPA in the initial phase of lysis. Our experiments confirmed the PLT dependent release rate, and the release rate constant in the current mechanistic model was derived from the in vitro data (24).

For brevity, distribution results for the rest of the scenarios are omitted here and can be found in Section C.2 of the Supporting Information.

#### Temporal Profiles of Averaged Concentrations over Clot Volume

**Fig. 7:**
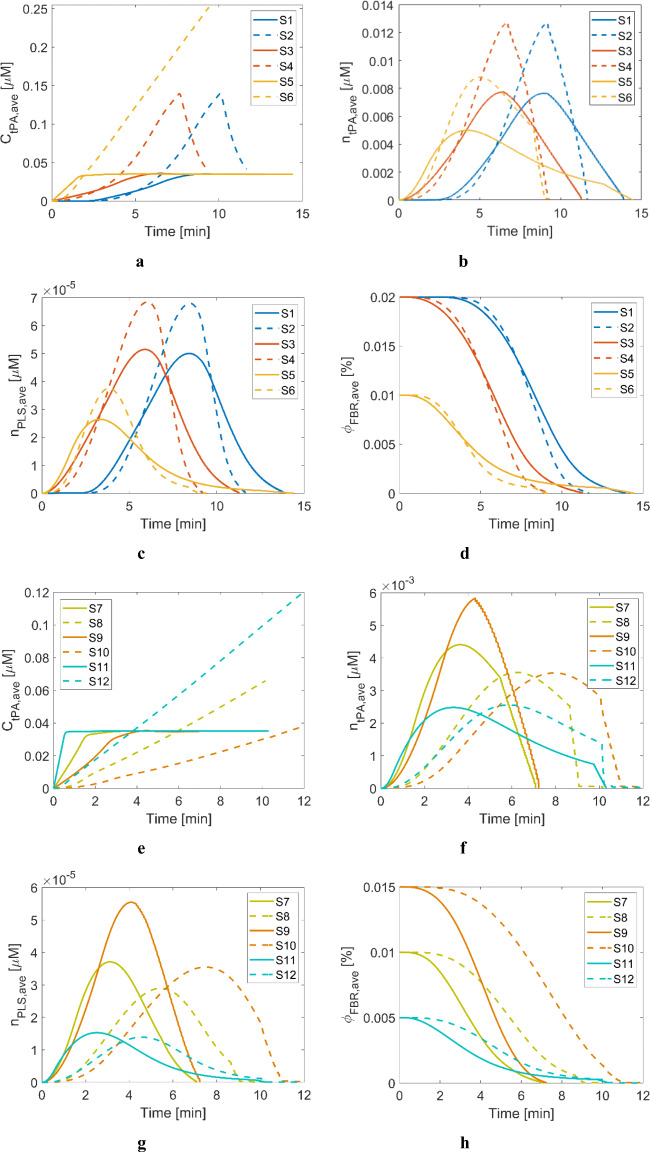
Averaged variables over the remaining clot volumes for Scenarios 1 to 12. (**a**) free tPA concentration for S1–6, (**b**) bound phase tPA concentration for S1–6, (**c**) bound phase PLS concentration for S1–6, (**d**) FBR volume fraction for S1–6, (**e**) free tPA concentration for S7–12, (**f**): bound phase tPA concentration for S7–12, (**g**) bound phase PLS concentration for S7–12, (**h**) FBR volume fraction for S7–12. Solid lines: treated with tPA, dashed lines: treated with tPA-loaded NV.

Simulation results demonstrate that recanalisation times are highly dependent on the clot location for treatment with both tPA and tPA-loaded NV, as reported in many clinical and computational studies (29, 32, 39). A distal clot takes a longer time to dissolve completely because the rate of convective drug transport is highly limited by a low permeation velocity in a blocked artery due to the high resistance imposed by a clot. The impact of a distal clot is clearly demonstrated in Fig. 7a, where the blue curves for S1-S2 are almost flat within the first 2 min since there is only consumption of plasma tPA in the clot phase due to FBR binding. The level of tPA in the clot starts to rise when the administered tPA reaches the clot front.

Interestingly, dense clots in S1–6 are dissolved more rapidly by NV than tPA, whereas coarse clots in S7–12 are more responsive to tPA than NV, as shown in Fig. 3a. This can be explained by the temporal profiles of free and bound tPA for S1–6 and S7–12 depicted in Fig. 7a-b and Fig. 7e-f, respectively. Both the free and bound phase tPA in the coarse clots are lower with NV due to slower tPA release resulting from fewer PLT present in the clots. Therefore, the curves for bound tPA and PLS in the coarse clots (S7–12) are shifted to the right with small peak values, compared to those in the dense clots (S1–6).

A general trend of slightly faster initial fibrinolysis with tPA than NV is observed in Fig. 7d and h for two reasons: firstly, the diffusivity of tPA is larger than that of NV, and hence tPA molecules move slightly faster than NV, as shown in Fig. 7a at around 2 min (for distal clots in S1 and S2). Secondly, the targeted thrombolytic treatment with NV has an additional step of triggered tPA release following NV binding to PLT. Therefore, there is a slight delay in initiating fibrinolysis, as clearly illustrated in Fig. 7d. The same observation was made in our experimental work, where the initial lysis with NV was delayed by approximately 2 min compared to tPA in a static setting (24). Nonetheless, it is seen in Fig. 7d that after the initial phase, the rate of lysis with NV (S2, S4 and S6) is much higher than that by tPA (S1, S3 and S5).

### Simulation Results with Varying Inlet Concentrations (S13 to S16)

Simulation results for S13-S16 are presented, with a focus on analysing the effects of different treatment protocols on systemic fibrinolytic proteins levels, spatially averaged concentration profiles and recanalisation times. Results of temporal and spatial variations obtained from the coupled systemic PKPD and local PD models are omitted in this section due to great similarity in the trends to S1-S12.

#### Results of Systemic PKPD Model

**Fig. 8:**
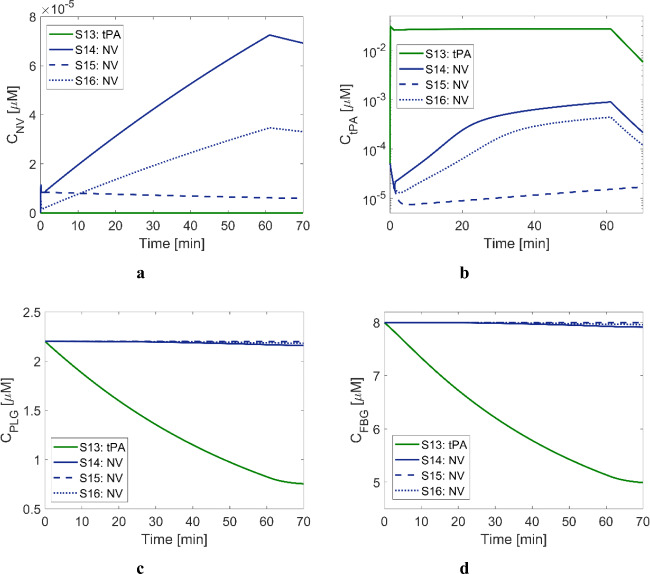
Simulation results of the systemic PKPD model for Scenarios 13 to 16. Temporal variations in concentrations of key species, (**a**) NV, (**b**) tPA, (**c**) PLG and (**d**) FBG. Solid green curve: treated with 0.9 mg/kg of tPA with the standard dosing regimen, solid navy curve: treated with 0.9 mg/kg of tPA-encapsulated NV with the standard dosing regimen, dashed navy curve: treated with 0.09 mg/kg of tPA-encapsulated NV in bolus, dotted navy curve: treated with 0.45 mg/kg of tPA-encapsulated NV with the standard dosing regimen (i.e., 10% of total dose in bolus + 90% in infusion).

Temporal variations in systemic levels of NV, tPA, PLG and FBG are presented in Fig. 8. For S14 and S16, where tPA-loaded NV is administered in two steps of bolus and continuous infusion, NV levels gradually increase over time, as shown in Fig. 8a. On the other hand, the tPA level in S13 is maintained at an almost constant level during the infusion over 1 h, depicted in Fig. 8b. The difference in the trends of circulating tPA and NV levels is attributable to their different half-life time and elimination rate. tPA has a very short half-life (4 min) and therefore is quickly excreted. On the other hand, the chosen half-life time for PEGylated NV is 132.61 min, about 30-fold larger than tPA, making NV reside within the body for much longer. In Fig. 8b, slightly elevated tPA levels for S14-S16 can be seen, compared to its initial systemic level (5 × 10^−5^ μM), despite the administration of NV only. This is due to passive leakage of tPA from NV by diffusion, as observed in our previous experimental studies (24). Owing to little systemic exposure of tPA for S14-S16, the levels of PLG and FBG are hardly affected by the systemic enzymatic reactions, unlike in S13 with tPA. Excessively fast conversion of PLG to PLS is not always favoured since this might slow down fibrinolysis in the later phase. Maintaining the systemic FBG level is a favourable characteristic of tPA-loaded NV, which can be linked to a reduced risk of systemic bleeding complications; 150 mg/dL (equivalent to approximately 4.4 μM) of FBG has been suggested as a threshold value for an increased risk of bleeding (40). Since plasma FBG levels vary over a wide range (5–13 μM) (41), it can be anticipated that some patients will suffer from bleeding complications following standard thrombolytic treatment, based on Fig. 8d.

#### Coupled Systemic PKPD and Local PD Simulation Results

**Fig. 9:**
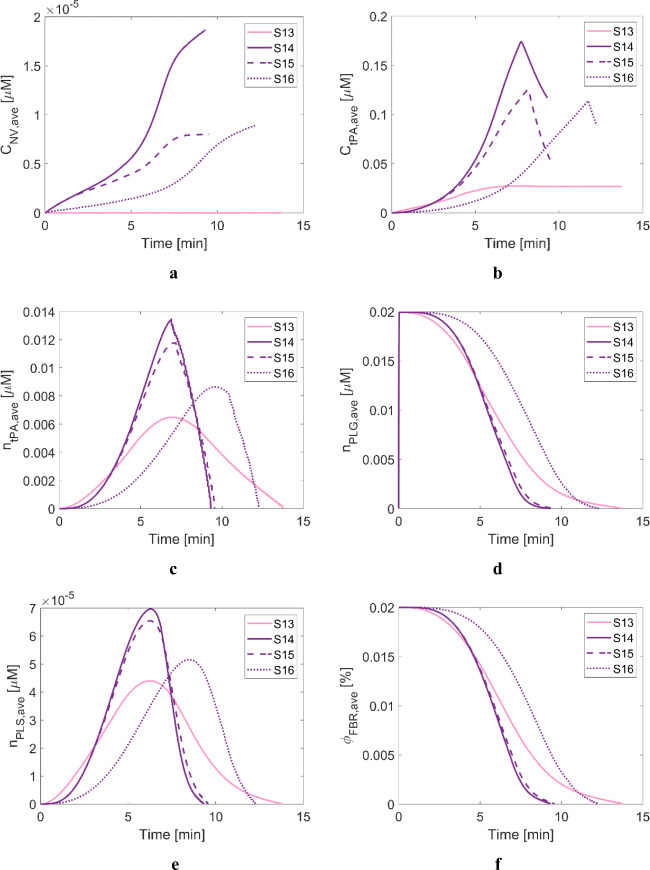
Averaged variables over the remaining clot volumes for Scenarios 7 to 10. (**a**) NV concentration, (**b**) free tPA concentration, (**c**) bound phase tPA concentration, (**d**) bound phase PLG concentration, (**e**) bound phase PLS concentration and (**f**) FBR volume fraction. Solid pink curve: treated with 0.9 mg/kg of tPA with the standard dosing regimen, solid purple curve: treated with 0.9 mg/kg of tPA-encapsulated NV with the standard dosing regimen, dashed purple curve: treated with 0.09 mg/kg of tPA-encapsulated NV in bolus, dotted purple curve: treated with 0.45 mg/kg of tPA-encapsulated NV with the standard dosing regimen (i.e., 10% of total dose in bolus + 90% in infusion).

The targeted thrombolytic treatment with the developed NV is not inferior to the standard treatment procedure with tPA in terms of shorter recanalisation times, as shown in Fig. 3b, i.e., 13.75 min with tPA vs 9.3–12.25 min with NV. Since all four scenarios have the same clot properties, differences in the results can mainly be attributed to the selected dosing protocols. As can be seen in Fig. 9b and c, free and bound tPA levels in the treatments with NV (S14-S16) are generally higher than that with tPA, except at the very beginning of the treatment. Consequently, bound PLG is rapidly converted to bound PLS, as illustrated in Fig. 9d and e.

Interestingly, there is no noticeable discrepancy in recanalisation time between S14 and S15, even though S15 uses only 10% of the total dose in the standard regimen. Despite a much lower dose used in S15, the NV level is comparable to S14 at up to 2 min and much higher than S16, as shown in Fig. 9a. As a result, such temporal profiles of bound tPA, PLG and PLS as in Fig. 9c-e are observed, respectively. Although the NV level in S14 drastically increases up to 1.9 × 10^–5^ μM, almost two-fold higher than S15 (8.0 × 10^–5^ μM), the difference in bound PLS between S14 and S15 is not pronounced. Also unlike the bolus-only protocol in S15, protocols with a combined bolus and continuous infusion (S14 and S16) exhibit a slower increase in the plasma levels of NV (Fig. 8a). In other words, the transport rate of NV into the occluded artery and resulting tPA release is rather low for infusion over a long time period (1 h), compared to the same dose in a bolus (5 s).

When comparing S14 and S16 with the same dosing protocol except for the total dose, the standard regimen in S14 appears to be superior to S16 in terms of shorter recanalisation time. Due to the lower infusion rate in S16, the initial concentrations of NV, tPA and bound phase species are low. The simulated scenarios have a relatively small clot with a length of 4 mm that can be dissolved in a fairly short time window, where lysis time is mainly governed by the initial levels of NV and tPA during the course of treatment. However, this does not necessarily mean that a high dose is favoured to achieve fast clot dissolution because high tPA levels are associated with depleted PLG and reduced lytic efficacy, as addressed earlier. Several studies also demonstrate that other dosing regimens might be more beneficial than the standard therapeutic regimen; Tebbe et al. reported that a single bolus of 50 mg of tPA for the treatment of acute myocardial infarction could achieve about 3-fold higher peak tPA level than the standard regimen (42). Furthermore, using a low dose of 0.6 mg/kg resulted in a comparable treatment outcome without an increased incidence of bleeding (43). Our simulations results (Fig. 3b) suggest that for the tPA-loaded NV, a bolus of 10% recommended tPA dose would be as effective as the standard regimen with the full recommended dose for the simulated clot. This has important implications for the potential use of our tPA-loaded NV as a new thrombolytic therapy that requires a significantly lower dose of tPA.

### Limitations and Future Scope

The multiphysics model of targeted thrombolytic therapy developed in this study is a versatile tool that helps us understand the complex mechanisms of targeted drug delivery and thrombolysis and predict the treatment outcomes with detailed information on varying clot properties and species levels in the body. However, there are several limitations in the current modelling approach that need to be addressed.

First of all, there is an intrinsic limitation of 1D models for blood flow and species transport without accounting for the effect of secondary motion caused by geometric variations, such as bifurcation, vessel curvature and partial occlusion. As a result, the 1D model can only be used to simulate occlusive clots in a straight arterial segment. For occlusive clots, it has been confirmed by several studies that clot size is one of the most critical factors that influence recanalisation time (44–46). Moreover, within smaller arteries such as cerebral arteries, occlusive clots are common (47–50). Hence there are numerous clinically relevant thrombolysis scenarios that can be simulated with the 1D model, which is particularly useful when evaluating the influence of various clot properties and treatment regimens. Given its advantage of being computationally more tractable than the 3D models developed in our previous studies (24, 29, 30), the 1D model can be used for model-based automated optimisation of treatment protocols.

Secondly, detailed experimental validations are lacking at this stage, especially *in situ* clot lysis images with the tPA-loaded NV. Our simulation results suggest that the critical extent of lysis with tPA-NV tends to start from the inside of the clot rather than from the clot front (Fig. 5). This would be very interesting if confirmed experimentally. In the same context, the simulated scenarios assume homogenous distributions of activated platelets and fibrin fibre within a clot. However, it is known that the cellular components of thrombi and emboli are not uniformly distributed within a clot (51–53); in general, clots have a denser core than the surroundings, i.e., higher PLT and FBR concentration at the centre. Moreover, the mobility of PLT within the clot is arbitrarily modelled via the hyperbolic tangent function, which results in a negligible reduction in PLT concentration in the clot. As a result, the current model possibly overpredicts the amount of released tPA, especially at the later phase of clot lysis, and subsequently underpredicts recanalisation time. It would be necessary to obtain *in situ* clot lysis images along with information on initial and time-varying clot structure and composition for thorough validation. This will also allow us to further tune the model parameters to suit a broader range of applications.

Finally, the systemic PKPD simulation results presented in Fig. 8 are based on a PK parameter value in the literature and are not yet confirmed by animal experiments with our tPA-loaded NV. PK parameters specific to our NV should be derived from animal experiments in the future. This will improve the credibility of simulation results and make it possible to use the model to search for an optimal dosing protocol of the tPA-loaded NV for efficient control of preclinical and further clinical studies.

## Conclusion

This work presents a multiphysics simulation model for thrombolysis via tPA and activated platelet targeted tPA-loaded NV. The computational model is based on 1D blood flow and species transport coupled with fibrinolytic reaction kinetics, targeted NV delivery and triggered tPA release in an occlusive blood clot composed of a fibrin fibre network and activated platelets. The systemic PKPD is also included in the model so that the circulating fibrinolytic proteins and drug levels can be used as inlet conditions for the simulated clot-containing artery. Our integrated simulation model is capable of predicting the progression of clot lysis and systemic FBG level, indicative of the bleeding risk, for different clinical scenarios.

The models of fibrinolytic reaction kinetics have already been validated against in vitro and clinical data, as described in our previous studies (28, 30). The mechanistic model for targeted tPA-NV delivery and tPA release rate along with the characteristic parameters of tPA-NV were derived from the *in vitro* experiments reported in our recent work (24). Nevertheless, further validations against well-controlled in vitro experiments are needed. It is hoped that our multiphysics simulation model can be used to optimise treatment regimens for existing and new thrombolytic therapies via benefit/risk assessment. Moreover, it provides a foundation for a virtual simulation platform aimed at supporting future preclinical and clinical trials, and the design of personalised therapy.

## Supplementary Information

ESM 1 (DOCX 11.6 mb)

## Data Availability

The datasets generated during and/or analysed during the current study are available from the corresponding author on reasonable request.
