# Supplementary material for: Multiphysics Modelling and Simulation of Thrombolysis via Activated Platelet-Targeted Nanomedicine
Source: Pharm Res. 2022 Jan 19;39(1):41–56. doi: 10.1007/s11095-021-03161-2 (PMC8837543; doi:10.1007/s11095-021-03161-2)
Supplement: Supplementary file 1 — (DOCX 11.6 mb) [file 11095_2021_3161_MOESM1_ESM.docx]

Supporting Information for

**Multiphysics modelling and simulation of thrombolytic therapy via** **activated platelet targeted nanomedicine**

Boram Gu^1,2^, Yu Huang^1,3^, Emily Louise Manchester^1^, Alun D. Hughes^4,5^, Simon A. McG. Thom^6^, Rongjun Chen^1^, Xiao Yun Xu^1,*^

^1^ Department of Chemical Engineering, Imperial College London, South Kensington Campus, London, United Kingdom

^2^ School of Chemical Engineering, Chonnam National University, Gwangju, Republic of Korea

^3^ Department of Radiology, Shanghai Jiao Tong University Affiliated Sixth People's Hospital, Shanghai Jiao Tong University School of Medicine, 600 Yi Shan Road, Shanghai, China

^4^ Institute of Cardiovascular Science, University College London, London, United Kingdom

^5^ MRC Unit for Lifelong Health and Ageing at University College London, London, United Kingdom

^6^ National Heart and Lung Institute, Imperial College London, London, United Kingdom

* Corresponding author: Xiao Yun Xu, yun.xu@imperial.ac.uk

**A. MODEL EQUATIONS**

Model equations are shown in this section. Parameter descriptions and their values are listed in Table B.1 to B.3 along with their sources. Additional modelling details and derivations can be found in our previous work [28-30].

**A.1 Clot properties**

*A.1.1 Initial clot properties*

Based on the initial volume fractions of fibrin fibre network (FBR) and activated platelets (PLT) in the clot, the following variables can be calculated:

Initial clot volume fraction: (A1)

Initial clot porosity: (A2)

Initial activated platelets concentration: (A3)

Initial fibrin binding site concentration: $n_{FBR,0}=\frac{2\times CS_{V}\times BS_{CS}}{N_{AV}},$ (A4)

$$\text{where }BS_{CS}=\sum_{i=1}^{N_{CS}+1} \frac{\pi}{sin^{-1}\frac{d\theta}{2idr}}, CS_{V}=\frac{LtVt}{0.5L_{M}}, LtVt=\frac{1}{\pi R_{f}^{2}}\frac{\rho_{fibrin}}{\rho_{fibre}} \text{ and }N_{CS}=\frac{R_{FBR}}{dr}$$

*A.1.2 Key variables related to time-varying clot properties*

Extent of lysis: $E_{L}=1-\frac{n_{tot}}{n_{tot,0}}$ (A5)

Extent of activated platelets present in the clot: $E_{PLT}=\frac{C_{PLT}}{C_{PLT,0}}$ (A6)

Fibrin fibre volume fraction: (A7)

Activated platelet volume fraction: (A8)

Fibrin fibre resistance: (A9)

Activated platelet resistance: (A10)

Clot resistance: $R_{clot,tot}=R_{clot,FBR}+R_{clot,PLT}$ (A11)

**A.2 Reaction kinetics**

In this work, thrombosis reactions are ignored and all the thrombolysis reactions included in the model are described below.

*A.2.1 Plasma reactions*

 (A12)

 (A13)

 (A14)

 (A15)

 (A16)

*A.2.2 Fibrinolytic reactions in the clot phase*

 (A17)

 (A18)

 (A19)

 (A20)

 (A21)

 (A22)

*A.2.3 Reaction between NV and INT*

 (A23)

 (A24)

 (A25)

 (A26)

By combining relevant reaction rates for each species, plasma reaction terms, *r_i_^plasma^* and *r_i_^clot^* in Eqs (1)-(4), (7)-(8) and (13)-(14) for *i* = tPA, NV, NV_emp_, PLG, PLS, AP, AP-PLS, FBG, MG, PAI, PLT-free, PLT-ND, PLT-NV_emp_, tPA-F, PLG-F, PLS-F, PLS-F_lysed_ and FBR can be formulated.

**A.3 Calculation of Flow in a Blocked Artery using Darcy’s Law**

The volumetric flowrate in Eq (6) is derived from the differential form of Darcy’s law:

$-\frac{\mu}{k_{clot}}u=\frac{dP}{dx}$ (A27)

By integrating it with the assumption of negligible pressure gradient in the clot-free area compared to that across the clot, the following equation is obtained:

$\int_{0}^{L} dP=-\int_{0}^{L} \frac{\mu}{k_{clot}}u dx=-\int_{0}^{L} \frac{\mu}{k_{clot}}\frac{Q}{A\varepsilon} dx$ (A28)

$u=\frac{\Delta P}{\mu\int_{0}^{L} \frac{R_{clot,tot}}{\varepsilon} dx}=\frac{\Delta P_{x}L_{clot,rem}}{\mu\int_{0}^{L} \frac{R_{clot,tot}}{\varepsilon} dx}$ (A29)

where ΔP is the pressure difference between the front face and back end of the clot. Δ*P_x_* is the pressure drop per unit length and *L_clot,rem_* is the remaining clot length.

**A.4 Calculation of Systemic PKPD Model Parameters**

Elimination constant: $k_{el,i}=\frac{\ln2}{t_{1/2,i}}$ (A30)

Generation rate: (A31)

**B. MODEL PARAMETERS AND SIMULATION CONDITIONS**

All the model parameters and simulation parameters are listed in the following tables for completeness. For the sources of the parameters, refer to our previous publications [24, 28-30], along with rationales for the selected values.

Table B.1 Kinetic parameters

| Symbol | Description | Value | Unit | Source |
| --- | --- | --- | --- | --- |
| *k_a,tPA_* | Adsorption coefficient for tPA | 0.01 | μM^-1^ s^-1^ | [S1] |
| *k_d,tPA_* | Desorption coefficient for tPA | 0.0058 | s^-1^ | [S1] |
| *k_a,PLG_* | Adsorption coefficient for PLG | 0.1 | μM^-1^ s^-1^ | [S2] |
| *k_d,PLG_* | Desorption coefficient for PLG | 3.8 | s^-1^ | [S3] |
| *k_a,PLS_* | Adsorption coefficient for PLS | 0.1 | μM^-1^ s^-1^ | [S2] |
| *k_d,PLS_* | Desorption coefficient for PLS | 0.05 | s^-1^ | [S2] |
| *K_M_* | Michaelis constant for PLG conversion in the bound phase | 0.1 | μM | [S4] |
| *k_cat_* | Michaelis reaction rate coefficient for PLG conversion in the bound phase | 0.3 | s^-1^ | [S5] |
| *k_deg_* | Lysis coefficient | 2.178 | s^-1^ | [S5] |
| *1/γ* | Cuts needed for PLS to cut 1 fibrin unit | 10 | - | [S2] |
| *k_a,NV_* | Adsorption coefficient for NV | 2.6224×10^-2^ | μM^-1^ s^-1^ | [24] |
| *k_d,NV_* | Desorption coefficient for NV | 7.5159×10^-3^ | s^-1^ | [24] |
| *k_rel_* | Triggered release rate constant | 0.10975 | s^-1^ | [24] |
| *K_leak_* | Leakage rate constant | 2.946×10^-9^ | s^-1^ | [24] |
| *K_M,PLG_* | Michaelis constant for PLG conversion in the free phase | 28.03 | μM | [S6] |
| *k_cat,PLG_* | Michaelis reaction rate coefficient for PLG conversion in the free phase | 0.3 | s^-1^ | [S6] |
| *K_M,FBG_* | Michaelis constant for FBG conversion in the free phase | 55 | μM | [S6] |
| *k_cat,FBG_* | Michaelis reaction rate coefficient for FBG conversion in the free phase | 250 | s^-1^ | [S6] |
| *k_AP,f_* | Forward reaction constant for AP and PLS | 10 | μM^-1^ s^-1^ | [S6] |
| *k_AP,r_* | Reverse reaction constant for AP and PLS | 0.0021 | s^-1^ | [S6] |
| *k_cat,AP_* | Inactivation rate constant by AP | 0.004 | s^-1^ | [S6] |
| *k_MG_* | Reaction constant for MG and PLS | 0.35 | μM^-1^ s^-1^ | [S7] |
| *k_PAI_* | Reaction constant for tPA and PAI | 37 | μM^-1^ s^-1^ | [S8] |
| *ν_rel_* | Stochiometric coefficient for tPA and NV reaction | 3,570 | - | [24] |

Table B.2 Parameters for estimating clot properties

| Symbol | Description | Value | Unit | Source |
| --- | --- | --- | --- | --- |
| *N_INT_* | Number of integrins expressed upon activation of platelets | 80,000 | numbers | [S9] |
| *P_max_* | Max. number of platelets per unit volume | 6.67×10^13^ | numbers | [S10] |
| *L_M_* | Length of fibrin monomer | 45 | nm | [S11] |
| *dr* | Protofibril inter-spacing in r-direction | 10 | nm | [S12] |
| *dθ* | Protofibril inter-spacing in *θ*-direction | 10 | nm | [S12] |
| *S_p_* | Sphericity of platelets | 1 | - | [S13] |
| *D_p_* | Platelet diameter | 2 | μm | [S13] |
| *E_L,crit_* | Critical extent of lysis | 0.95 | - | [S12] |
| *R_FBR_* | Fibrin fibre radius | 60 | nm | [S11] |

Table B.3 Transport model parameters

| Symbol | Description | Value | Units | Source |
| --- | --- | --- | --- | --- |
| *C_AP,0_* | Initial AP concentration | 1 | µM | [S11] |
| *C_FBG,0_* | Initial FBG concentration | 8 | µM | [S11] |
| *C_PLG,0_* | Initial PLG concentration | 2.0 | µM | [S11] |
| *C_PLS,0_* | Initial PLS concentration | 0 | µM | [S11] |
| *C_tPA,0_* | Initial tPA concentration | 0.05×10^-3^ | µM | [S11] |
| *C_MG,0_* | Initial MG concentration | 3 | μM | [S11] |
| *C_PAI,0_* | Initial PAI concentration | 5.23 × 10^−4^ | μM | [S14] |
| *t_1/2,AP_* | Half life time of AP | 2.64 | day | [S15] |
| *t_1/2,FBG_* | Half life time of FBG | 4.14 | day | [S16] |
| *t_1/2,PLG_* | Half life time of PLG | 2.2 | day | [S17] |
| *t_1/2,PLS_* | Half life time of PLS | 0.1 | s | [S18] |
| *t_1/2,tPA_* | Half life time of tPA | 4 | min | [S19] |
| *t_1/2,PAI_* | Half life time of PAI | 1.5 | hr | [S20] |
| *t_1/2,MG_* | Half life time of MG | 3 | min | [S21] |
| *t_1/2,NV_* | Half life time of NV | 132.61 | min | [25] |
| *V_c_* | Plasma volume | 3.9 | L | [S17] |
| *M_w,tPA_* | tPA molecular weight | 59.04 | mg/µmol | - |
| *M_w,NV_* | NV molecular weight | *v_rel_*×*M_w,tPA_* | mg/µmol |  |
| ∆*P_x_* | Pressure drop per unit length across the clot | 60 | mmHg/cm | [S11] |
| *D_a_* | Diameter of occluded artery | 3 | mm | [S22] |
| *μ* | Blood viscosity | 0.0037 | Pa∙s | [S23] |
| *D_proteins_* | Diffusivity of plasma proteins | 5×10^-11^ | m^2^/s | [S24] |
| *D_NV_* | Diffusivity of NV | 1.6×10^-12^ | m^2^/s | [35] |
| *D_PLT_* | Diffusivity of PLT | 3.1×10^-14^ | m^2^/s | [36] |

**C. ADDITIONAL SIMULATION RESULTS**

**C.1 Systemic PKPD model results**

Scenarios with tPA-loaded NV (S14-S16) show negligible decreases in FBG, AP and MG, compared to S13 with free tPA. This is due to the very low level of tPA present in the plasma phase as a result of tPA leakage from NV. Also the level of PAI slowly decreases over time for NV (S14-16), compared to tPA (S13). This suggests the encapsulation of tPA in NV can protect the inhibitory action of PAI on tPA activity.

**Fig C1** Scenario 13: the standard dosing regimen with conventional tPA (a total dose of 0.9 mg/kg with 10 % as a bolus and 90 % as a continuous infusion over 1hr)

**Fig C2** Scenario 14: the standard dosing regimen with tPA-loaded NV (an equivalent total dose of 0.9 mg/kg with 10 % as a bolus and 90 % as a continuous infusion over 1hr )

**Fig C3** Scenario 15: a bolus of tPA-loaded NV (an equivalent total dose of 0.09 mg/kg, 10% of the standard dose)

**Fig C4** Scenario 16: the standard dosing procedure with tPA-loaded NV (an equivalent total dose of 0.45 mg/kg, 50% of the standard dose, with 10 % as a bolus and 90 % as a continuous infusion over 1hr).

**C.2 Spatial and temporal variations of various model variables**

Figs C5 to C14 are spatial and temporal distributions of key species for S1-8 with constant inlet concentrations and S13-14 with time-varying inlet concentrations. Since patterns are very similar for the same clot properties and type of drug, selected cases are included here. Analysis can be found in the main manuscript.


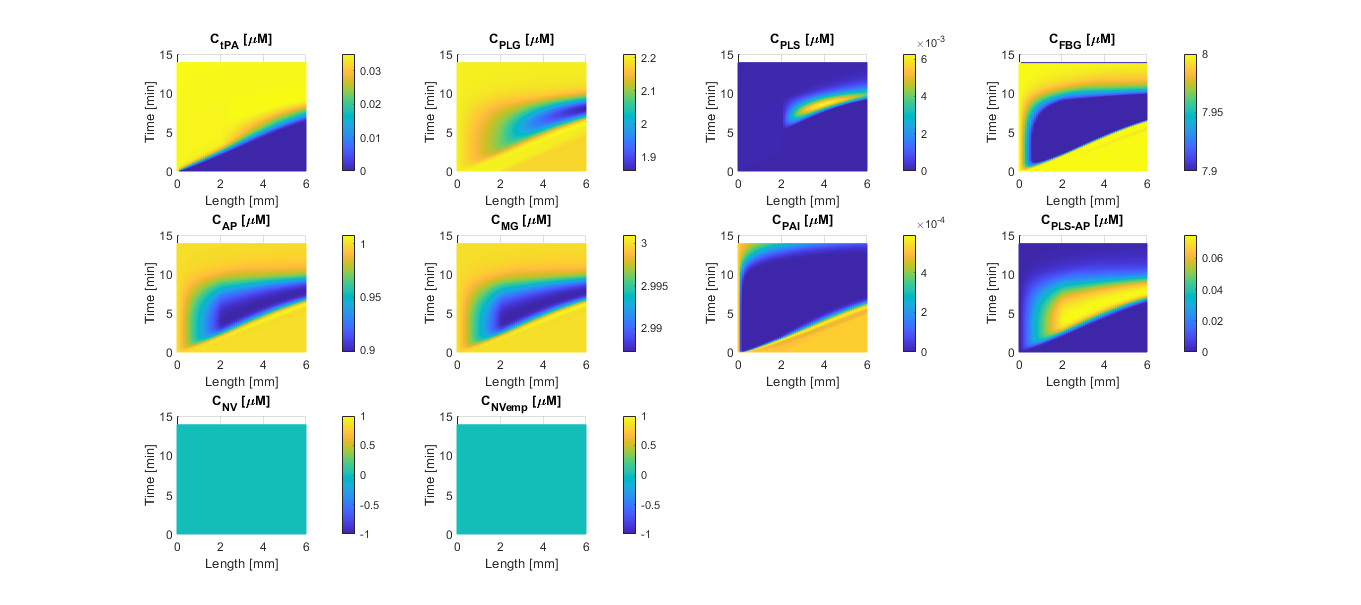


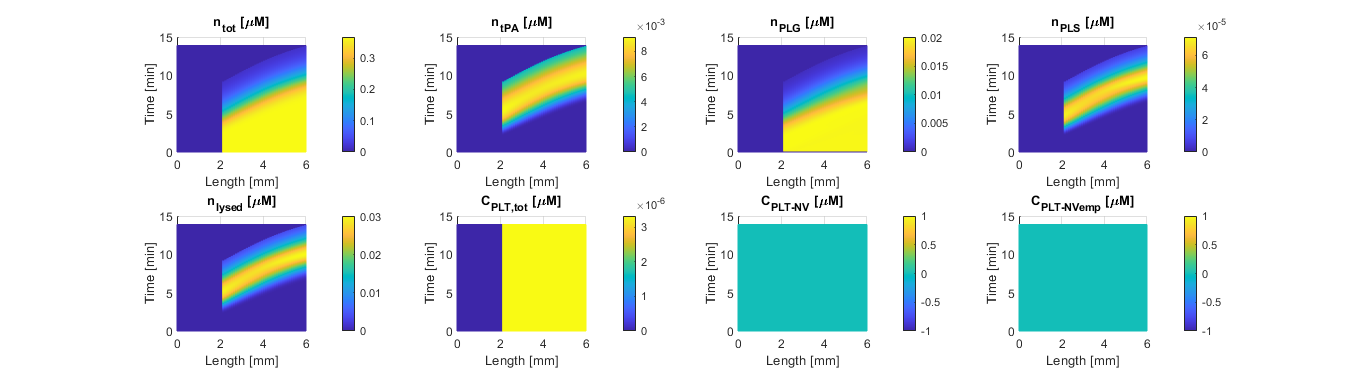

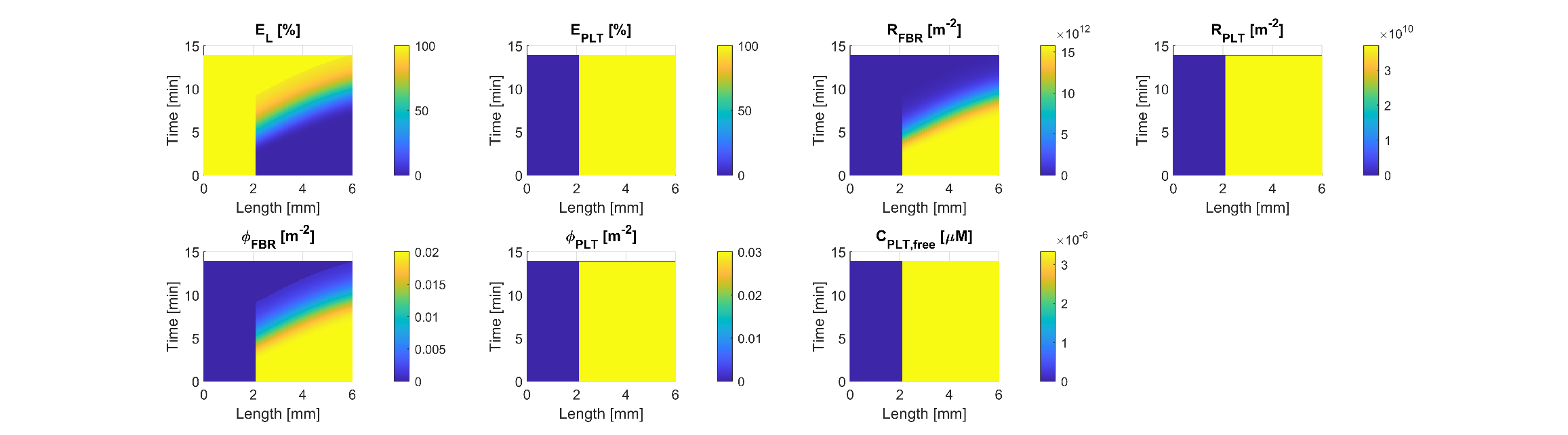


**Fig C5** Simulation results of Scenario 1: a 4-mm clot with *ϕ_FBR_* = 0.02 and *ϕ_PLT_* = 0.03 is located 2 mm away from the entrance of the blocked artery and is treated with free tPA. Fixed inlet concentrations of drug and plasma proteins are used.

**
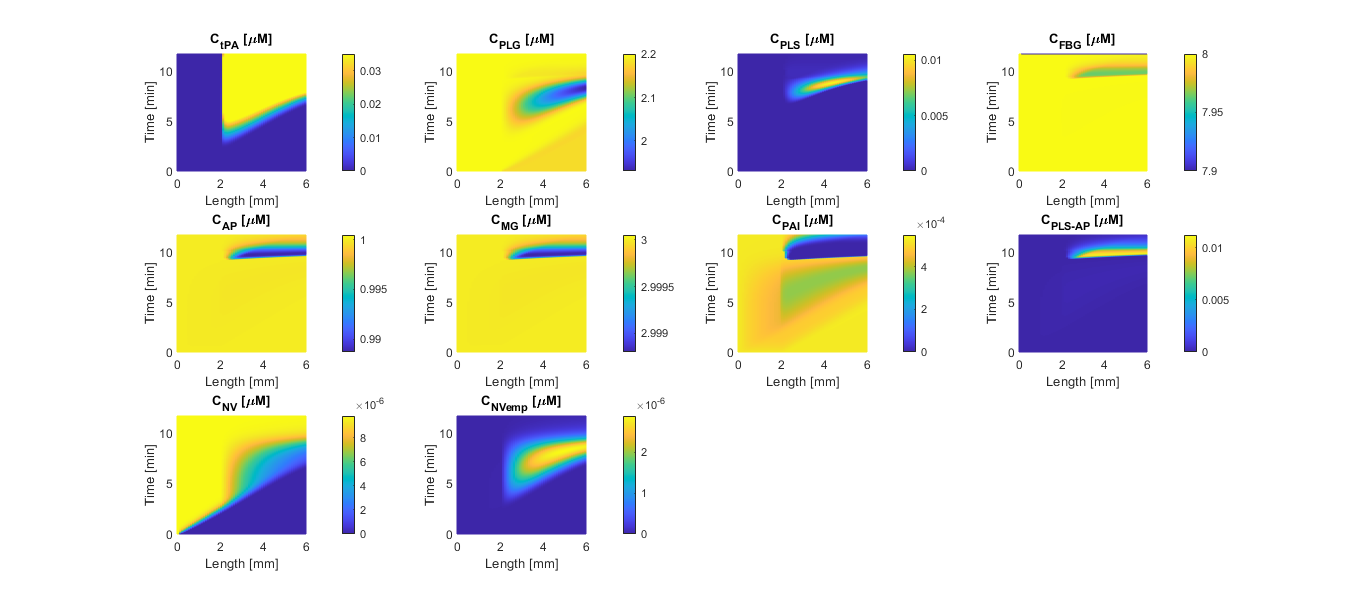
**


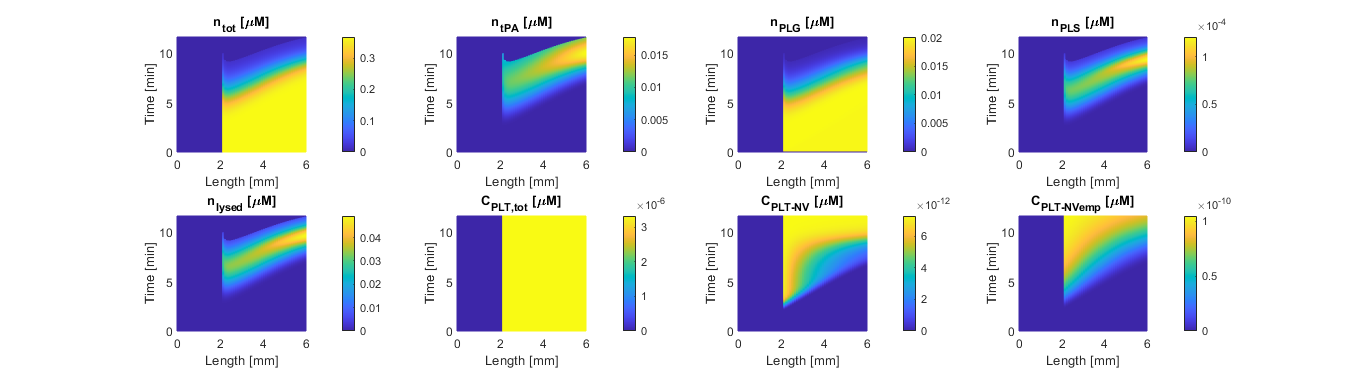


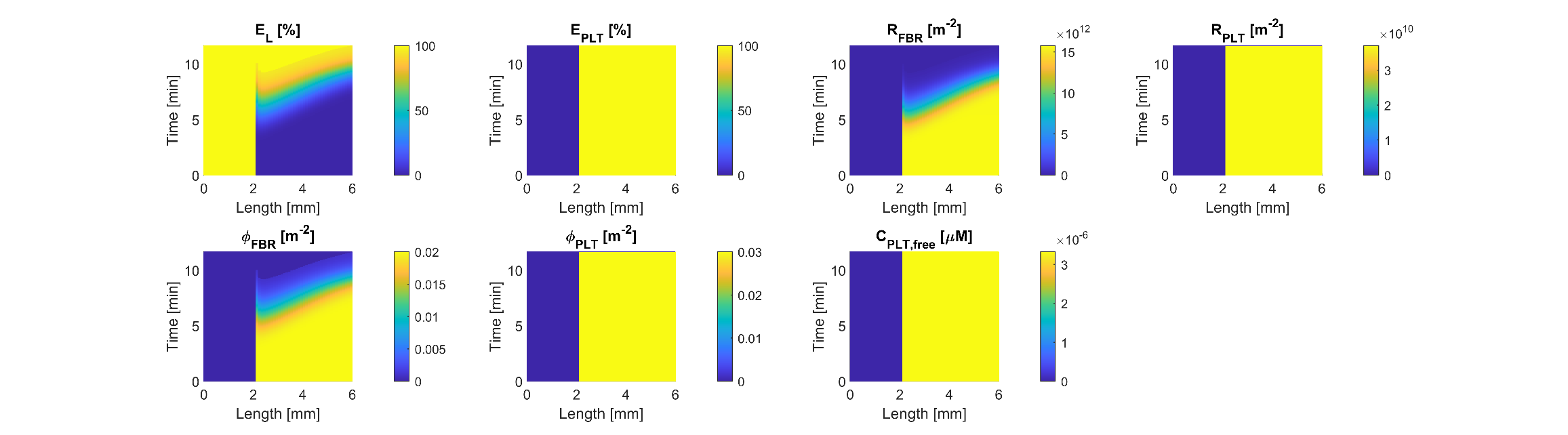


**Fig C6** Simulation results of Scenario 2: a 4-mm clot with *ϕ_FBR_* = 0.02 and *ϕ_PLT_* = 0.03 is located 2 mm away from the entrance of the blocked artery and is treated with NV. Fixed inlet concentrations of drug and plasma proteins are used.

**
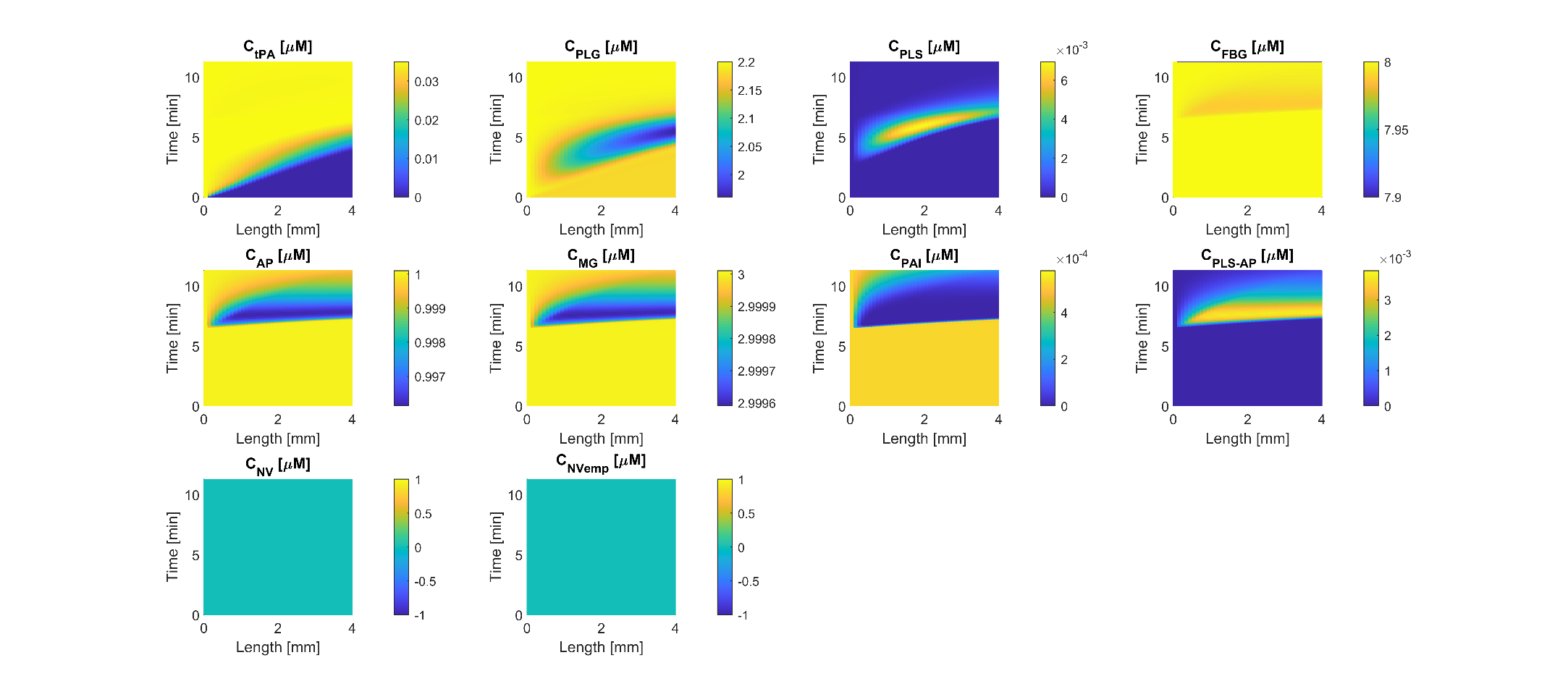
**


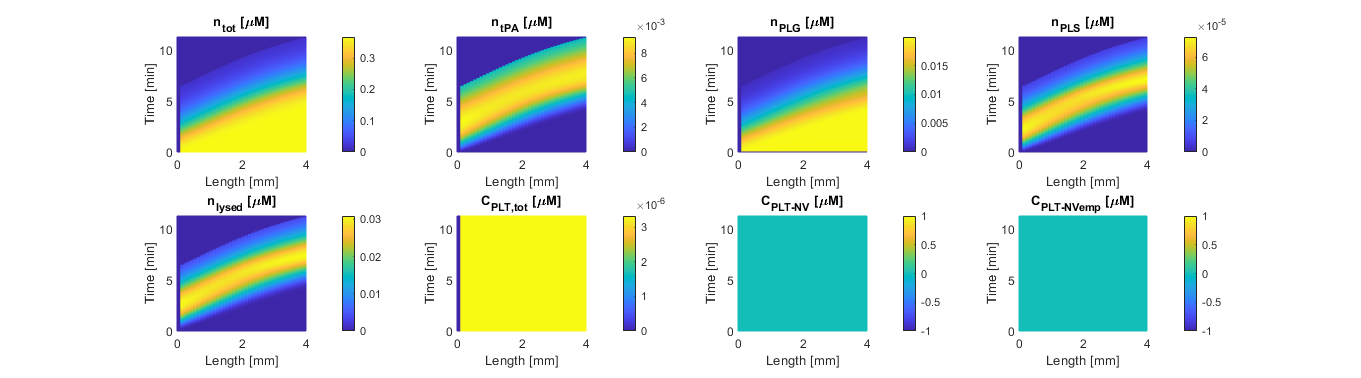

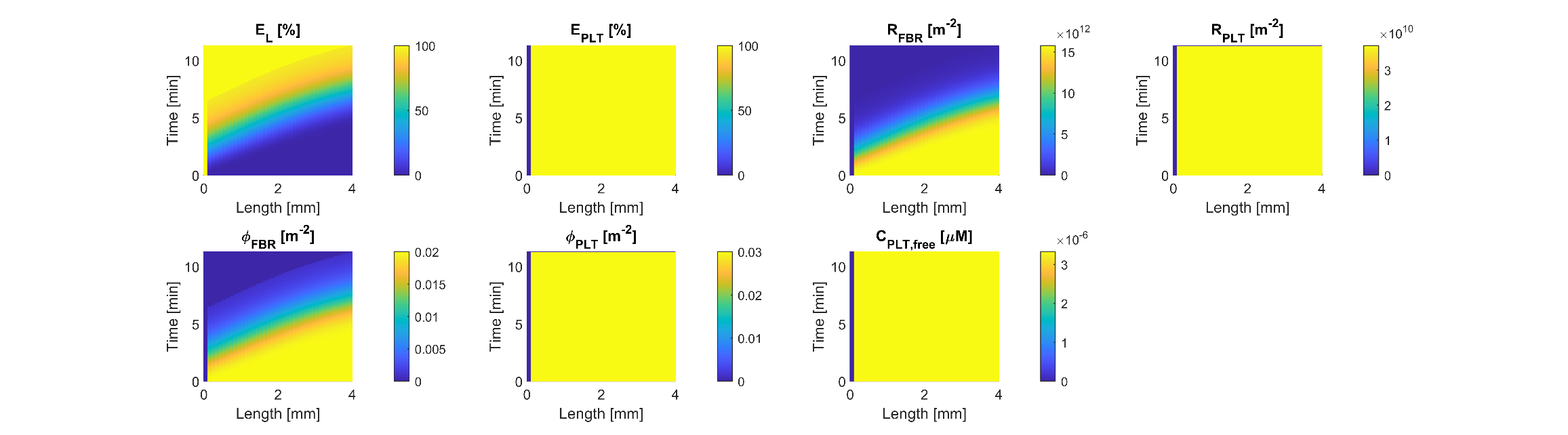


**Fig C7** Simulation results of Scenario 3: a 4-mm clot with *ϕ_FBR_* = 0.02 and *ϕ_PLT_* = 0.03 is located at the entrance of the blocked artery and is treated with free tPA. Fixed inlet concentrations of drug and plasma proteins are used.


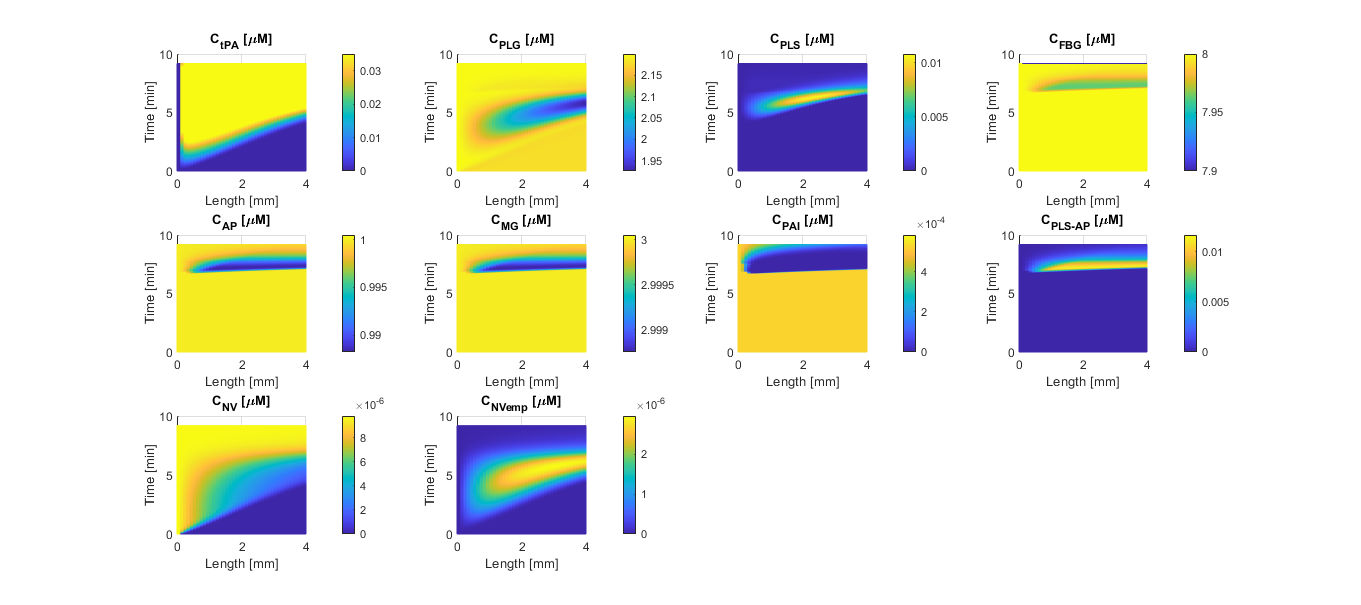


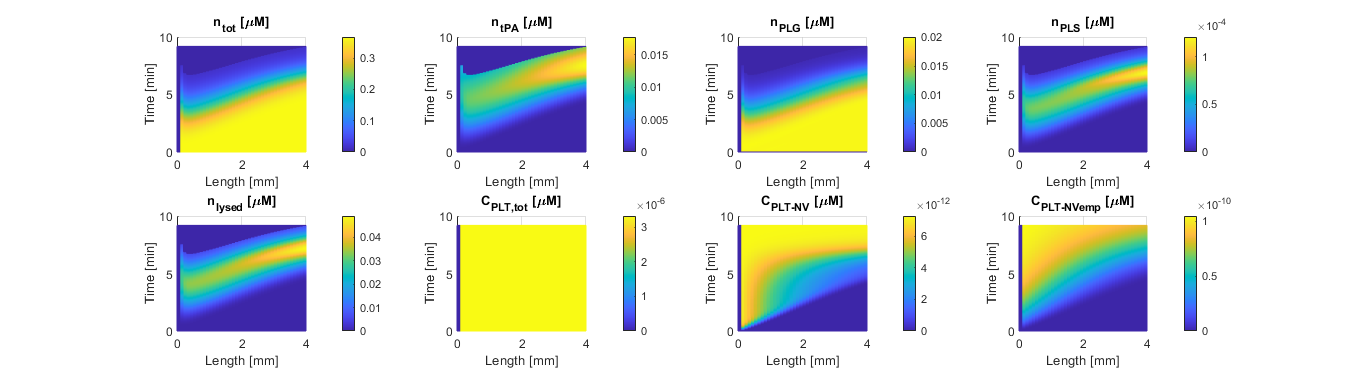

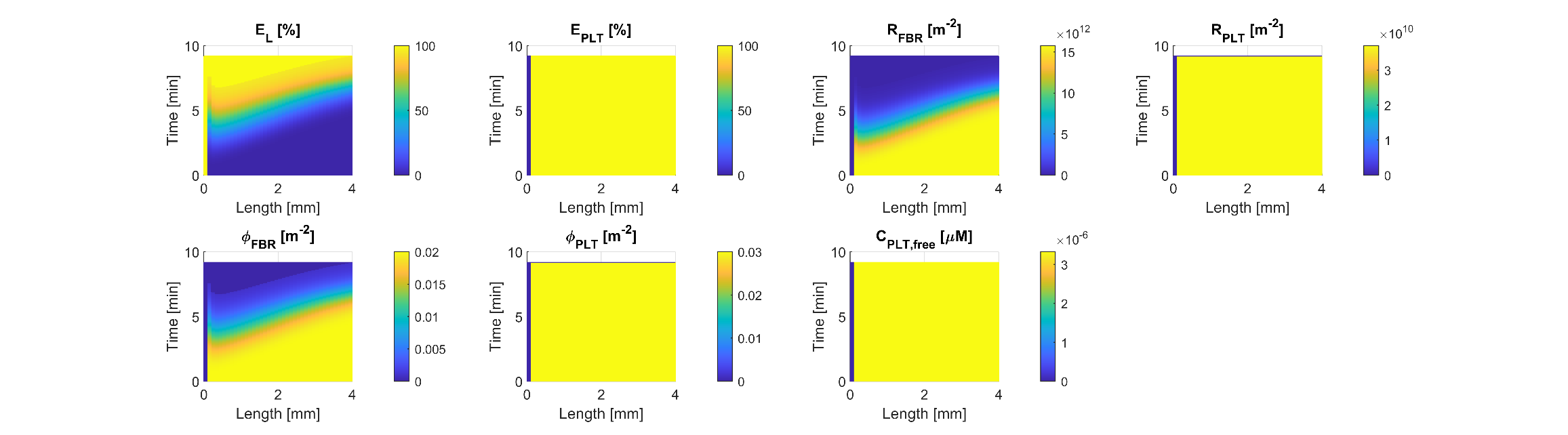


**Fig C8** Simulation results of Scenario 4: a 4-mm clot with *ϕ_FBR_* = 0.02 and *ϕ_PLT_* = 0.03 is located at the entrance of the blocked artery and is treated with NV. Fixed inlet concentrations of drug and plasma proteins are used.


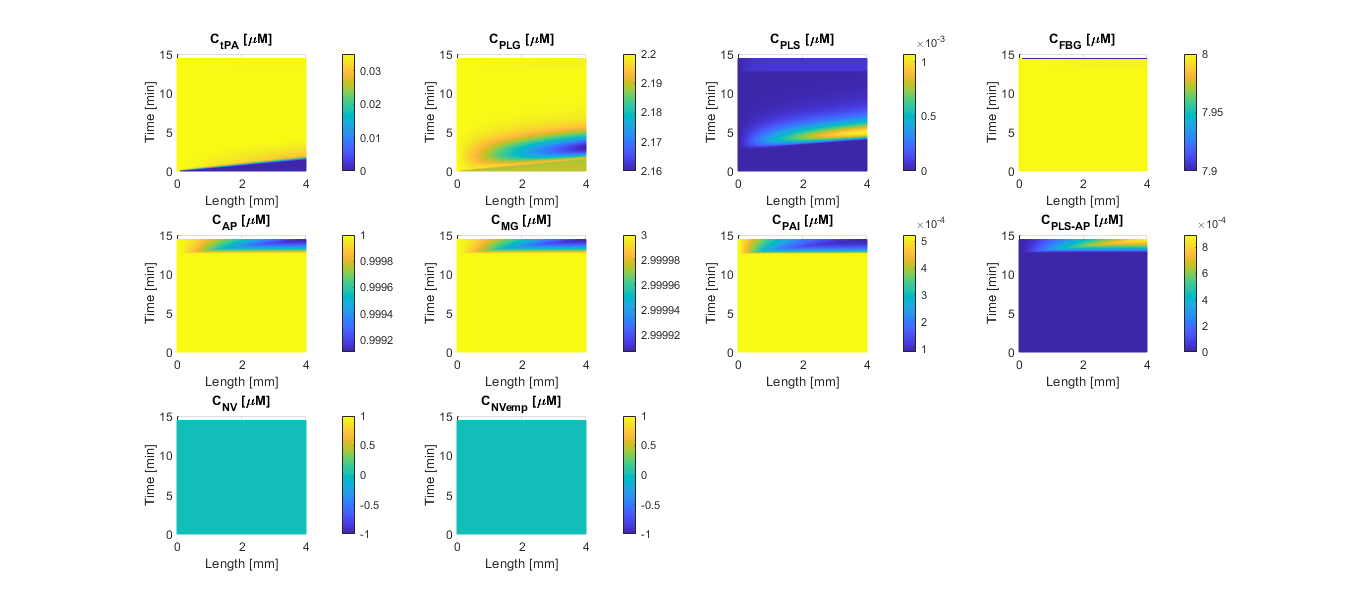


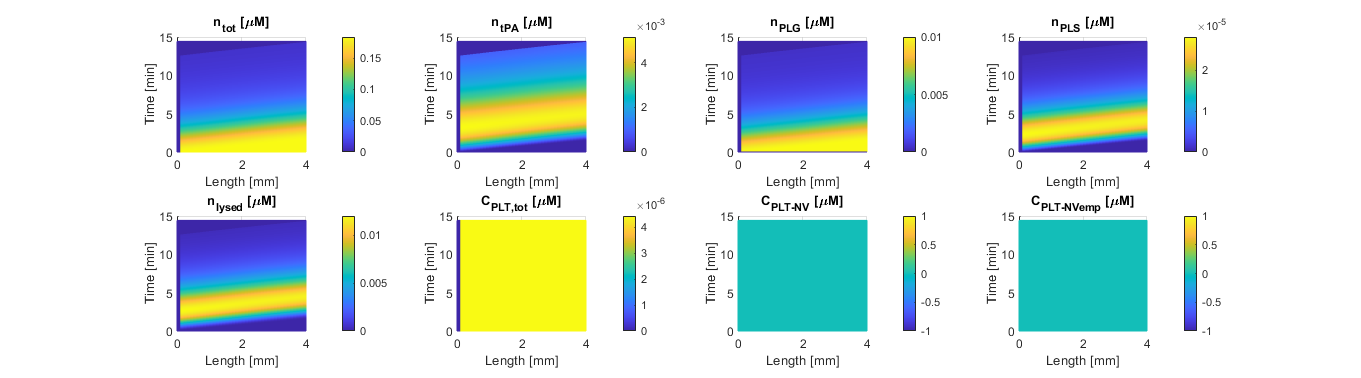

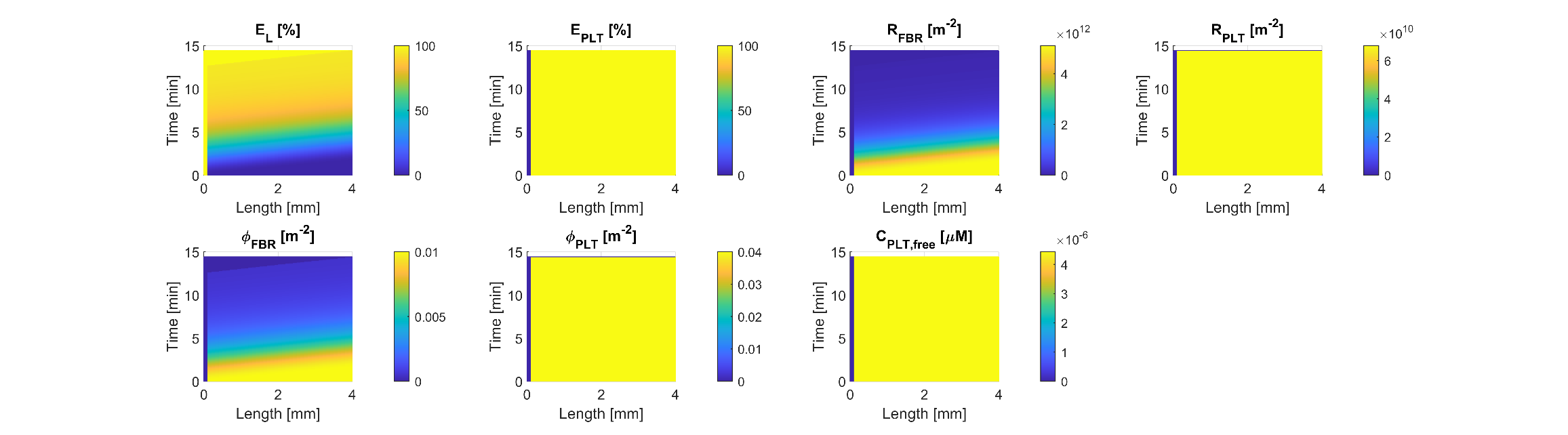


**Fig C9** Simulation results of Scenario 5: a 4-mm clot with *ϕ_FBR_* = 0.01 and *ϕ_PLT_* = 0.04 is located at the entrance of the blocked artery and is treated with tPA. Fixed inlet concentrations of drug and plasma proteins are used.


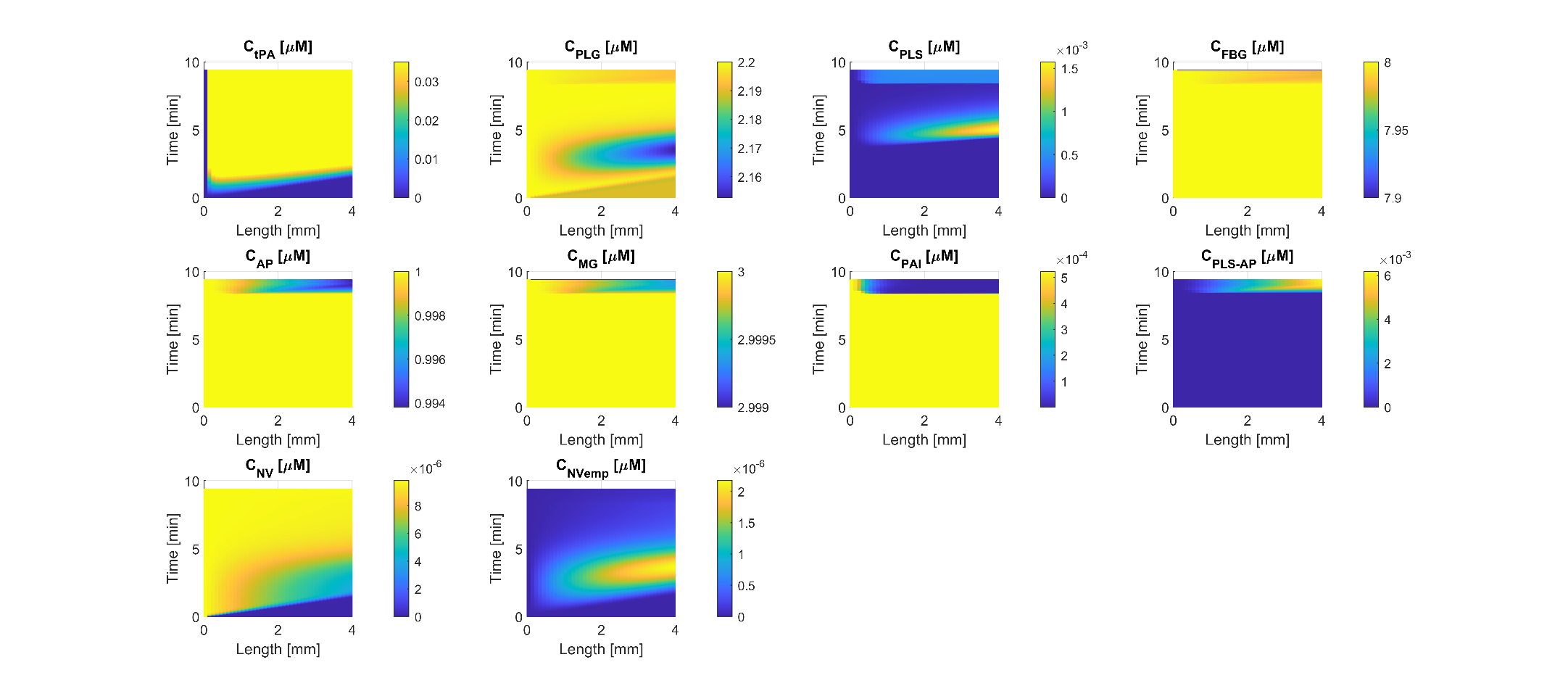


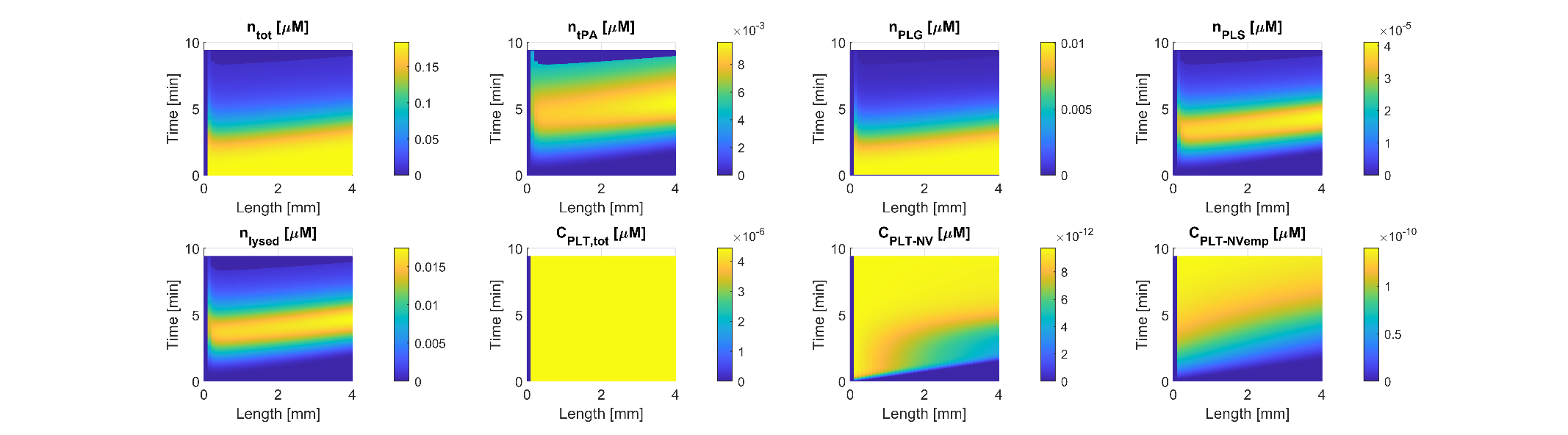

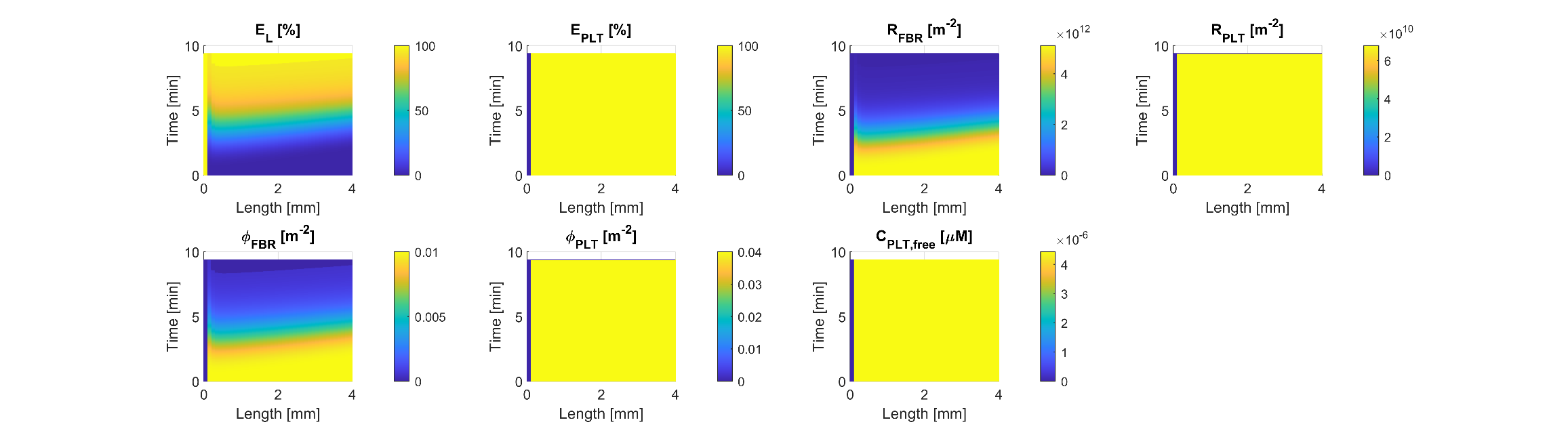


**Fig C10** Simulation results of Scenario 6: a 4-mm clot with *ϕ_FBR_* = 0.01 and *ϕ_PLT_* = 0.04 is located at the entrance of the blocked artery and is treated with NV. Fixed inlet concentrations of drug and plasma proteins are used.


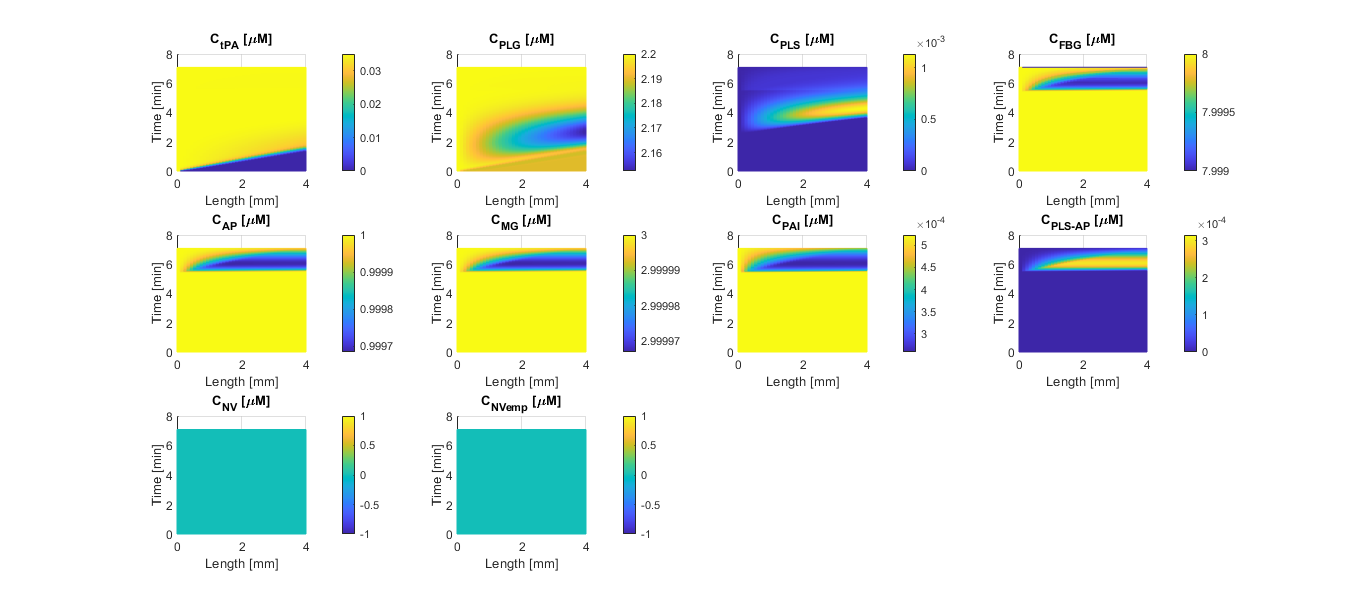

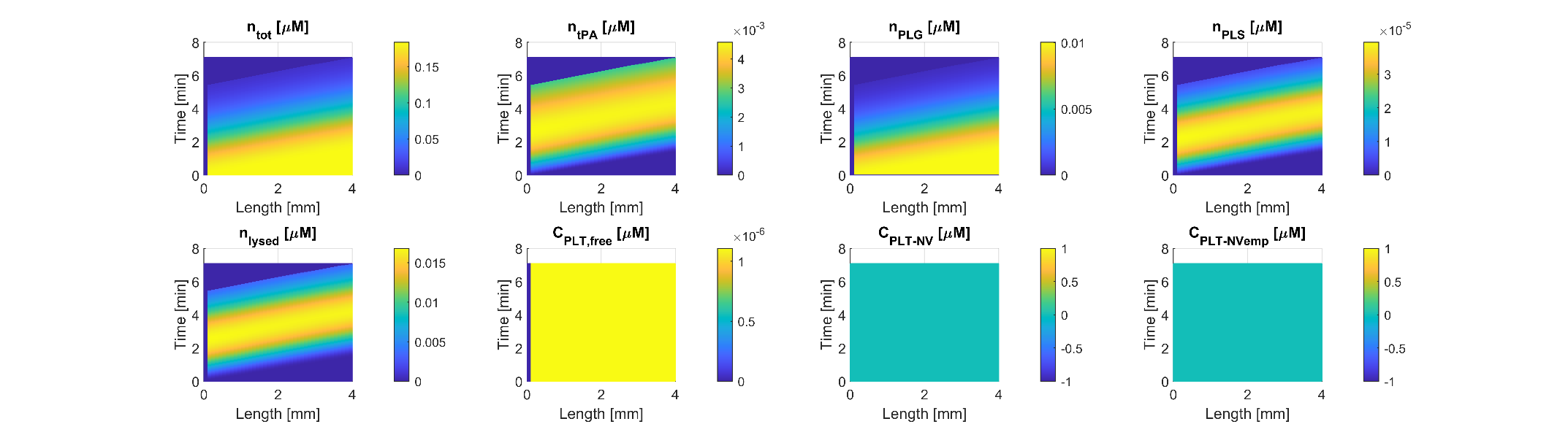

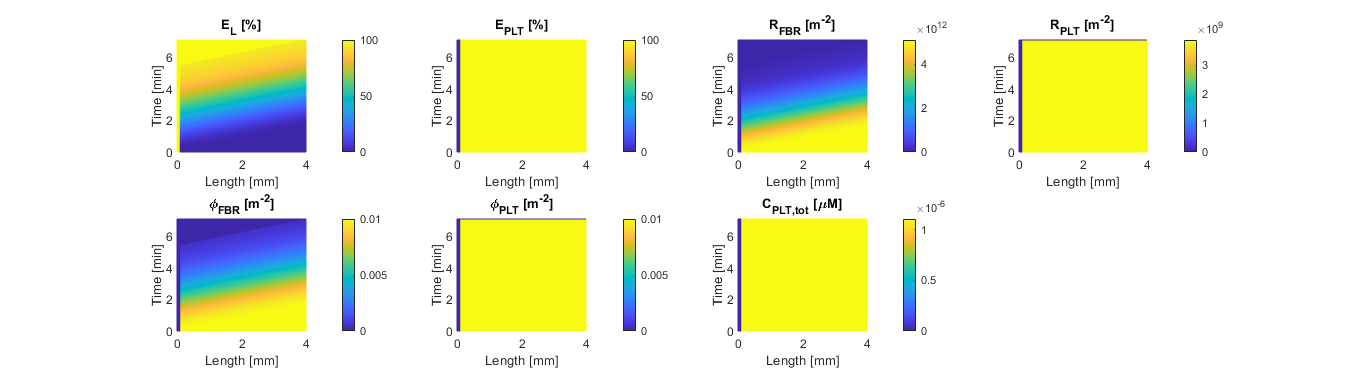


**Fig C11** Simulation results of Scenario 7: a 4-mm clot with *ϕ_FBR_* = 0.01 and *ϕ_PLT_* = 0.01 is located at the entrance of the blocked artery and is treated with tPA. Fixed inlet concentrations of drug and plasma proteins are used.


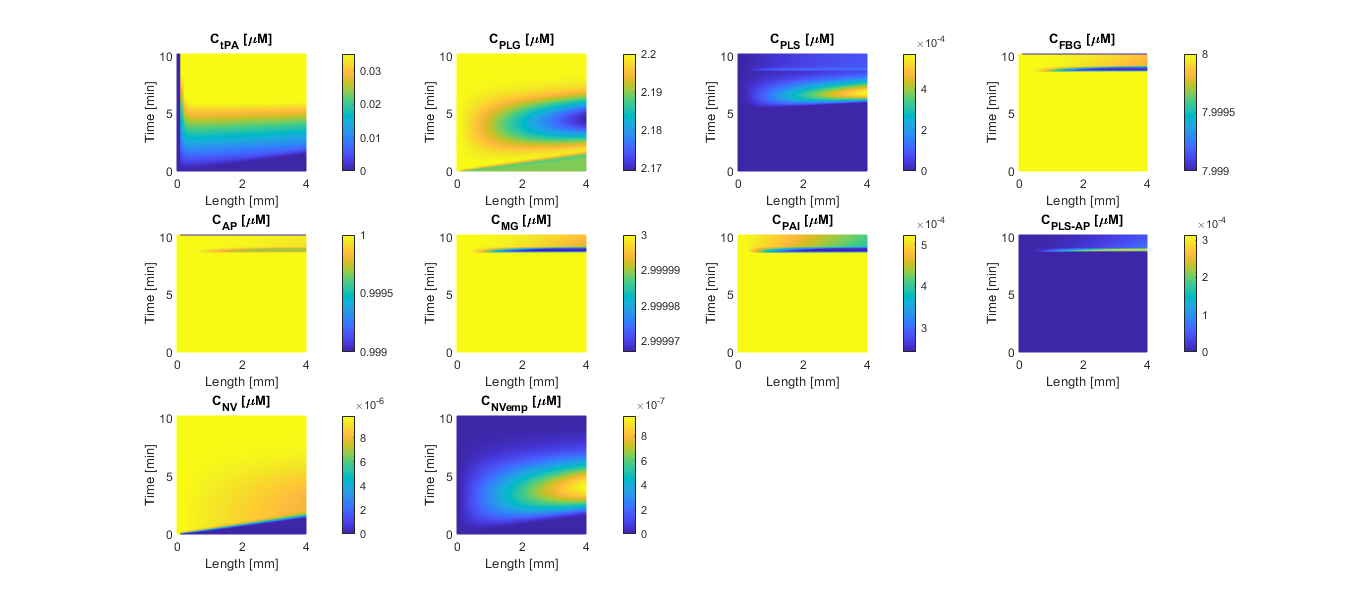

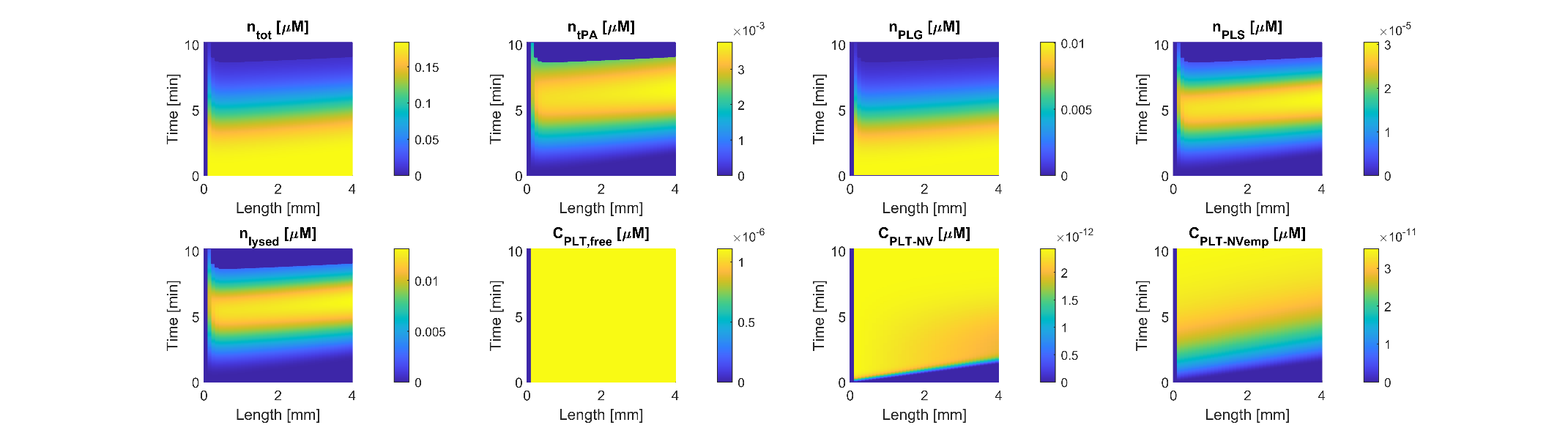

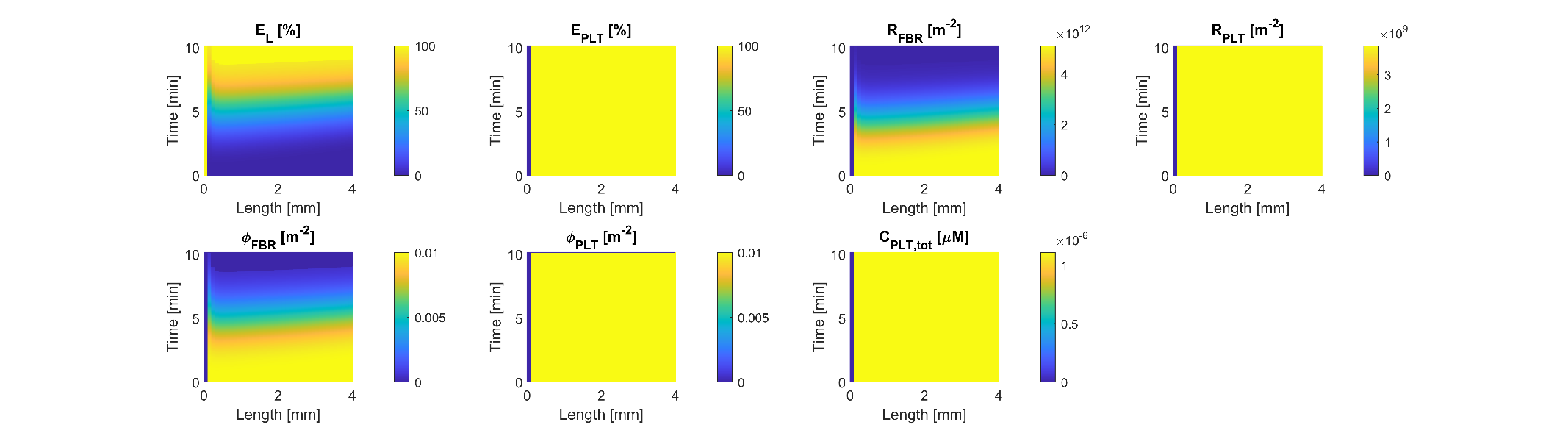


**Fig C12** Simulation results of Scenario 8: a 4-mm clot with *ϕ_FBR_* = 0.01 and *ϕ_PLT_* = 0.01 is located at the entrance of the blocked artery and is treated with NV. Fixed inlet concentrations of drug and plasma proteins are used.

**
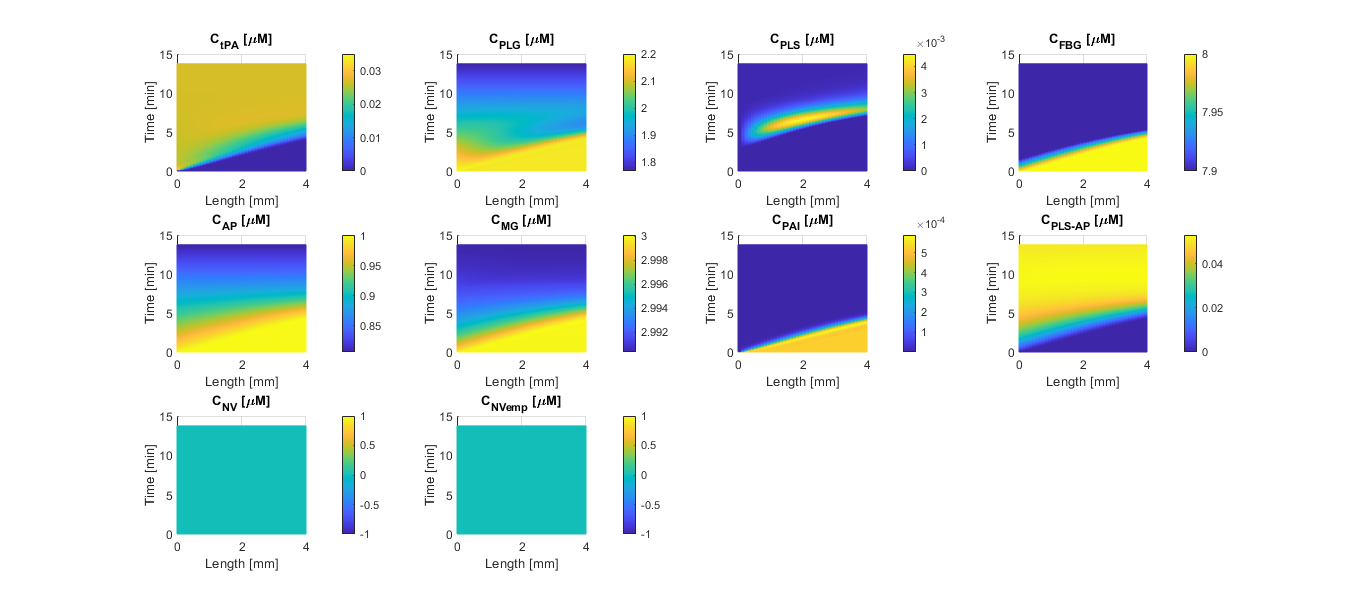
**

**
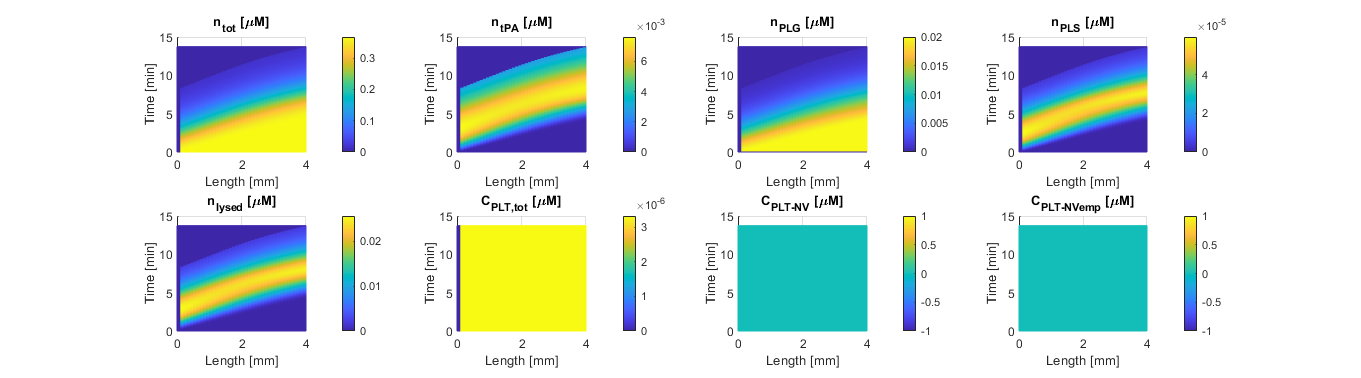

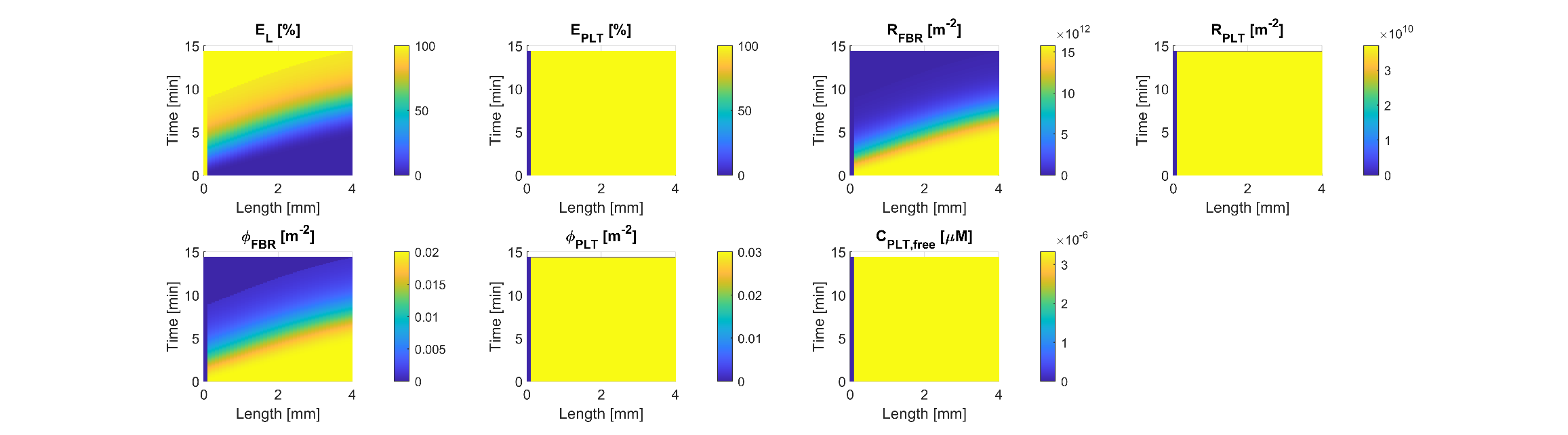
**

**Fig C13** Simulation results of Scenario 13: a 4-mm clot with *ϕ_FBR_* = 0.02 and *ϕ_PLT_* = 0.03 is located at the entrance of the blocked artery and is treated with free tPA. Time-varying inlet concentrations of drug and plasma proteins are used, obtained from the systemic PKPD model with the standard dosing regimen (a total dose of 0.9 mg/kg with 10% bolus and 90% continuous infusion).

**
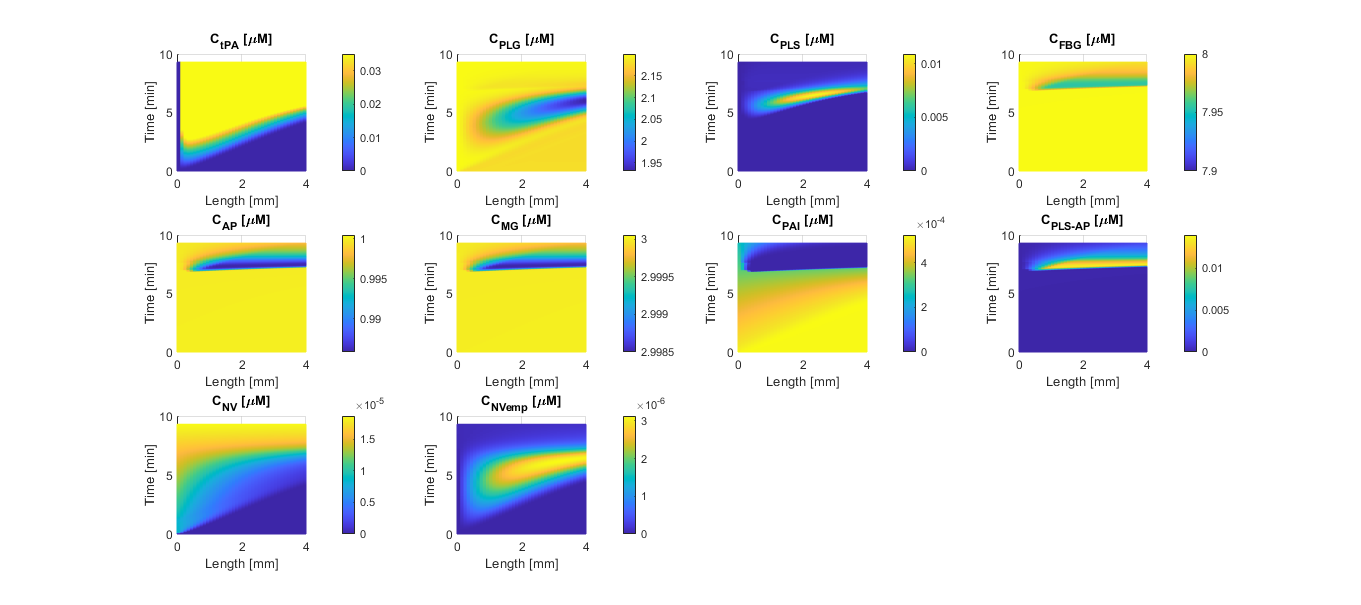
**

**
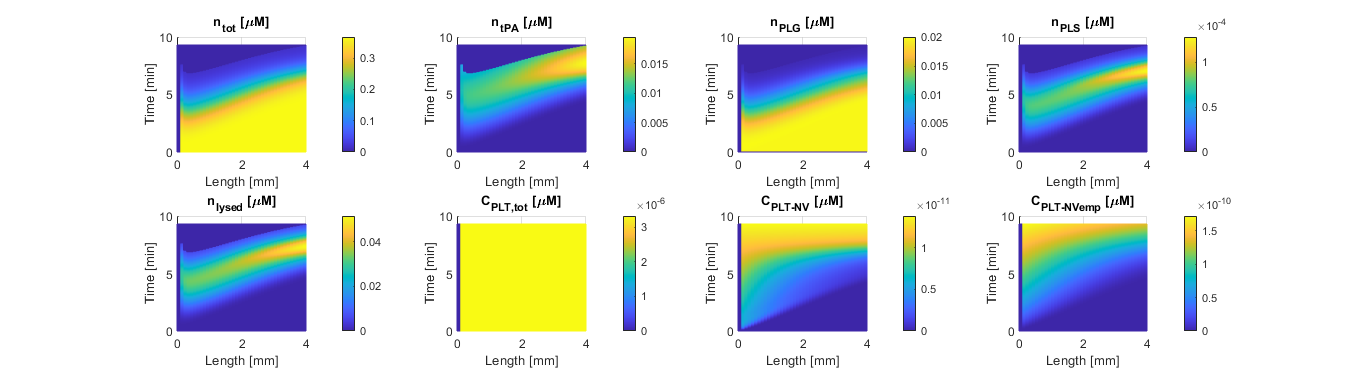

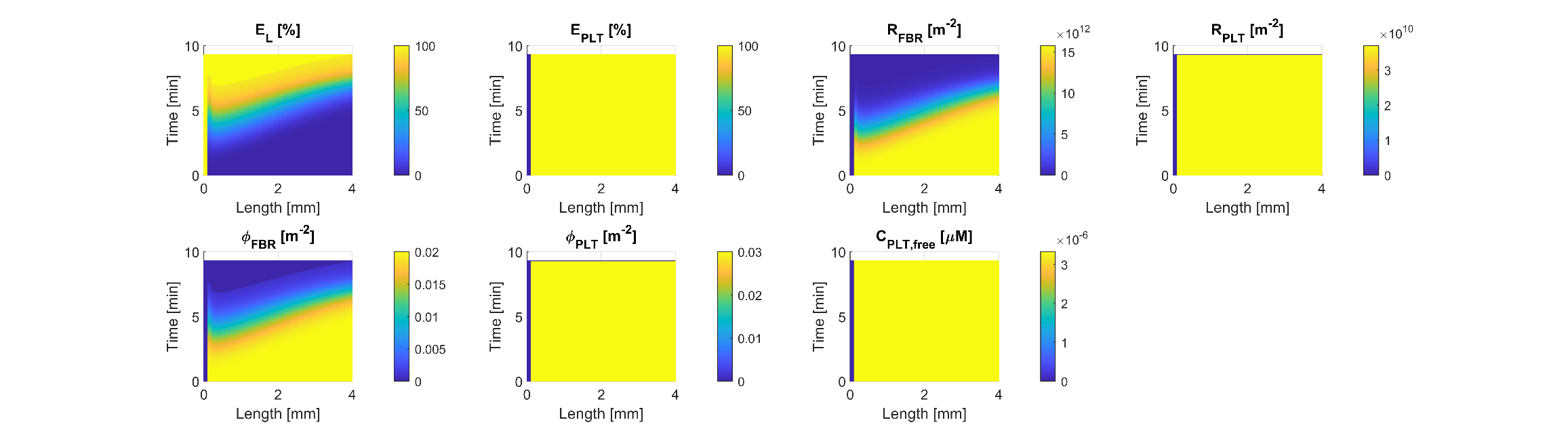
**

**Fig C14** Simulation results of Scenario 14: a 4-mm clot with *ϕ_FBR_* = 0.02 and *ϕ_PLT_* = 0.03 is located at the entrance of the blocked artery and is treated with NV. Time-varying inlet concentrations of drug and plasma proteins are used, obtained from the systemic PKPD model with the standard dosing regimen (0.9 mg/kg of encapsulated tPA with 10% bolus and 90% continuous infusion).

**C.3 Effects of the mobility function of platelets (PLT) M(t) in Eq. (9)**

Since the mobility function is arbitrarily chosen for all simulations (*m*=10), it is worth investigating the influence of the mobility coefficients on simulation results. To do so, we repeat the simulation for Scenario 4 with different values of *m* in the mobility function, as shown in Fig C15.

Fig C15. Comparison of different PLT mobility functions.

Predicted lysis times for different mobility functions are summarised below:

- *m* =10 (used for the original simulation) lysis time of 9.19 min
- *m*= 5 lysis time of 9.19 min
- *m* = 3 lysis time of 9.37 min

With smaller values of *m*, activated platelets tend to be more mobile even at low extents of lysis. Several key variables for *m*=3 are shown in Fig C16. Major differences are observed in variables related to platelets; due to higher mobility at *m*=3, platelets start to move axially even at a lower extent of lysis, hence delayed drug release and fibrinolysis.


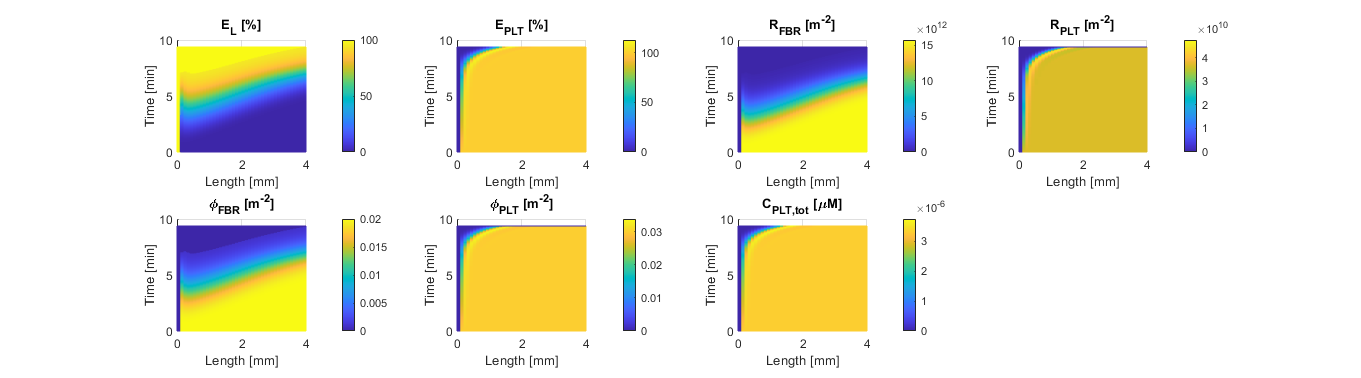


Fig C16: Simulation results when *m* = 3

As shown here, the PLT mobility function has an impact on lysis time and might even lead to incomplete lysis if activated platelets move out of the clot too quickly. In the future, a parameter sensitivity analysis will be carried out, in addition to experimental investigation to determine the mobility of activated platelets within the clot during clot lysis.

**References**

[S1] Husain, S. S., Hasan, A. A. & Budzynski, A. Z. Differences between binding of one-chain and two-chain tissue plasminogen activators to non-cross-linked and cross-linked fibrin clots. Blood. 1989;74:999–1006.

[S2] Wootton, D. M., Popel, A. S. & Rita Alevriadou, B. An experimental and theoretical study on the dissolution of mural fibrin clots by tissue‐type plasminogen activator. Biotechnol. Bioeng. 2002;77:405–419.

[S3] Lucas, M. A., Fretto, L. J. & McKee, P. A. The binding of human plasminogen to fibrin and fibrinogen. J. Biol. Chem. 1983;258:4249–4256.

[S4] Hoylaerts, M., Rijken, D. C., Lijnen, H. R. & Collen, D. Kinetics of the activation of plasminogen by human tissue plasminogen activator. Role of fibrin. J. Biol. Chem. 1982;257:2912–2919.

[S5] Kunitada, S., FitzGerald, G. A. & Fitzgerald, D. J. Inhibition of clot lysis and decreased binding of tissue-type plasminogen activator as a consequence of clot retraction. Blood. 1992;79:1420–1427.

[S6] Sobel, B.E.; Gross, R.W.; Robinson, A.K. Thrombolysis, clot selectivity, and kinetics. Circulation. 1984;70: 160–164.

[S7] Tiefenbrunn, A.J.; Graor, R.A.; Robison, A.K.; Lucas, F.V.; Hotchkiss, A.; Sobel, B.E. Pharmacodynamics of tissuetype plasminogen activator characterized by computer-assisted simulation. Circulation. 1986;73:1291–1299.

[S8] Chandler, W.L.; Alessi, M.C.; Aillaud, M.F.; Henderson, P.; Vague, P.; Juhan-Vague, I. Clearance of tissue plasminogen activator (TPA) and TPA/plasminogen activator inhibitor type 1 (PAI-1) complex: Relationship to elevated TPA antigen in patients with high PAI-1 activity levels. Circulation. 1997;96:761-768.

[S9] S. J. Joo, Mechanisms of platelet activation and integrin αIIbβ3. Korean Circ J. 2012;42:295-301.

[S10] K. Leiderman, Grow with the flow: a spatial-temporal model of platelet deposition and blood coagulation under flow. Math. Med. Biol. 2011;28:47-84.

[S11] S. L. Diamond, Engineering design of optimal strategies for blood clot dissolution. Annu. Rev. Biomed. Eng. 1999;1:427-461.

[S12] A. Piebalgs, B. Gu, D. Roi, K. Lobostesis, S. A. Thom, X. Y. Xu, Computational simulations of thrombolytic therapy in acute ischaemic stroke. Sci. Rep. 2018;8:15810.

[S13] A. R. Wufsus, N. E. Macera, K. B. Neeves, The hydraulic permeability of blood clots as a function of fibrin and platelet density. Biophys. J. 2013;104:1812-1823.

[S14] Vaughan, D.E. PAI-1 and atherothrombosis. J. Thromb. Haemost. 2005;3:1879–1883.

[S15] Collen, D. & Wiman, B. Turnover of antiplasmin, the fast-acting plasmin inhibitor of plasma. Blood. 1979;53:313–324.

[S16] Collen, D., Tytgat, G. N., Claeys, H. & Piessens, R. Metabolism and distribution of fibrinogen. I. Fibrinogen turnover in physiological conditions in humans. Br. J. Haematol. 1972;22:681–700.

[S17] Collen, D., Tytgat, G., Claeys, H., Verstraete, M. & Wallen, P. Metabolism of plasminogen in healthy subjects: effect of tranexamic acid. J. Clin. Investig. 1972;51:1310–1318.

[S18] Lijnen, R. & Collen, D. Protease inhibitors of human plasma. α-2-antiplasmin. J Med.1985;16:225–284.

[S19] Tanswell, P., Seifried, E., Su, P. C., Feuerer, W. & Rijken, D. C. Pharmacokinetics and systemic effects of tissue-type plasminogen activator in normal subjects. Clin. Pharmacol. Ther. 1989;46:155–162 .

[S20] Gorlatova, N.V.; Cale, J.M.; Elokdah, H.; Li, D.; Fan, K.; Warnock, M.; Crandall, D.L.; Lawrence, D.A. Mechanism of inactivation of plasminogen activator inhibitor-1 by a small molecule inhibitor. J. Biol. Chem. 2007; 282:9288–9296.

[S21] Jensen, P.E.H.; Humle Jørgensen, S.; Datta, P.; Sørensen, P.S. Significantly increased fractions of transformed to total α2-macroglobulin concentrations in plasma from patients with multiple sclerosis. Biochim. Biophys. Acta—Mol. Basis Dis. 2004;1690:203–207.

[S22] Gibo, H.; Carver, C.C.; Rhonton, A.L., Jr.; Carla, L.; Mitchell, R.J. Microsurgical anatomy of the middle

cerebral artery. J. Neurosurg. 1981;54:151–169.

[S23] Lowe, G. D. O. et al. Blood viscosity, fibrinogen, and activation of coagulation and leukocytes in peripheral arterial disease and the normal population in the Edinburgh Artery Study. Circulation. 1993;87:1915–1920.

[S24] Weigandt, K. M. et al. Fibrin clot structure and mechanics associated with specific oxidation of methionine residues in fibrinogen. Biophys. J. 2012;103:2399–2407.
